# Dynamic stability analysis of mixed-composition platoons with spatially weighted cooperative control

**DOI:** 10.1371/journal.pone.0342915

**Published:** 2026-03-27

**Authors:** Yulu Dai, Xueli Ge, Mingfeng Dai, Yanbin Liu, Aixi Yang

**Affiliations:** 1 Hangzhou Vocational & Technical College, Hangzhou, Zhejiang, China; 2 Anhui Province Key Laboratory of Intelligent Car Wire-Controlled Chassis System, Wuhu, Anhui, China; 3 Zhejiang Intelligent internet automobile innovation center, Hangzhou, Zhejiang, China; 4 Hangzhou Geely Automobile Co., Ltd., Hangzhou, Zhejiang, China; 5 Tsinghua University, Beijing, China; 6 Zhejiang University, Hangzhou, China; Beijing Institute of Technology, CHINA

## Abstract

In mixed traffic environments, the spatial distribution of Connected and Automated Vehicles (CAVs) plays a decisive yet previously unquantified role in platoon stability and safety. This study establishes a generalized stability modeling framework for heterogeneous platoons composed of Human-Driven Vehicles (HDVs), Autonomous Vehicles (AVs), and CAVs. By introducing a spatial weighting coefficient (γ), the proposed model captures the influence of longitudinal CAV positioning and allows for flexible representation of any vehicle composition pattern. Linear stability analysis and time-domain simulations are conducted to investigate the interaction between spatial distribution, communication delay, and dynamic response. The results demonstrate that front- and center-loaded CAV configurations effectively suppress velocity perturbations and maintain string stability even under moderate delay, while rear-loaded configurations exhibit early instability. Furthermore, an optimal γ range of 0.3–0.5 is identified to minimize oscillation amplitude, providing a practical guideline for cooperative control strategies in mixed platoons. The findings offer theoretical insights and quantitative evidence for optimizing CAV deployment to enhance stability and robustness in future intelligent transportation systems.

## 1. Introduction

Recent advancements in connected and automated vehicle (CAV) and automated vehicle (AV) technologies have the potential to significantly enhance traffic stability and safety [1–3]. On one hand, connected technologies enable vehicle-to-vehicle (V2V) and vehicle-to-infrastructure (V2I) communication, allowing vehicles to make more informed driving decisions based on real-time traffic data [4], while enabling traffic management centers to implement more effective control strategies. On the other hand, automation technology enhances driving performance by eliminating the unpredictability associated with human drivers, thereby improving the overall efficiency and orderliness of the traffic system [5,6].

Mixed traffic flow is a prominent area of contemporary research [7–9], largely driven by the relatively slow market penetration of CAV and AV, which hampers their seamless integration into existing transportation systems. In traditional traffic scenarios dominated by human-driven vehicles (HDV), drivers often face significant challenges in adapting to unconventional intersection designs and complex traffic regulations [10]. These challenges can result in diminished traffic efficiency and, in certain cases, pose safety risks. Furthermore, extensive investigations have been conducted on multi-class mixed traffic flows involving various combinations of vehicle types and control mechanisms, such as CAV and HDV [11–13], AV and HDV [14–16], and a mix of CAV, AV, and HDV [17,18].

In addition to differences in vehicle characteristics, CAVs exhibit unique traffic operational features that distinguish them from conventional vehicle types. A particularly salient aspect of CAV operation is platoon formation, which has been the subject of extensive research [19–21]. Numerous studies have demonstrated that platooning can significantly enhance traffic throughput and mitigate the amplification of traffic instabilities [22–24]. Moreover, a variety of factors associated with platoon dynamics have been thoroughly investigated in the literature, including platoon size, formation strategies, and platoon-based traffic flow models [25–27]. These studies contribute to a deeper understanding of the potential advantages and challenges of integrating platoon operation within mixed traffic environments, offering insights into how such systems might optimize overall traffic efficiency and stability. Standing in the wake of existing relevant literature [28,29], spatial distribution of vehicles—particularly CAV—in mixed platoons may influence platoon stability and safety. Notably, some studies have explored the spatial distribution of CAVs within platoons and its effects on platoon stability. For instance, Chen et al. (2025) [30] explored how CAV positioning modulates stability in mixed platoons. However, these studies largely focus on specific configurations or simplified models, and they do not fully account for the nonlinear interactions between vehicle types, particularly in mixed traffic scenarios with varied communication delays and dynamic control responses.

Recent studies have investigated the use of predictive control strategies for electric vehicle platoons, particularly in challenging environments such as hilly roads. These predictive strategies aim to optimize energy efficiency while maintaining platoon stability. Such approaches demonstrate how ecological considerations, such as energy consumption and terrain adaptation, can be integrated into cooperative control strategies for mixed platoons, providing a unique perspective for enhancing the performance of CAVs in diverse traffic environments.

This paper fills the gap by introducing a generalized stability modeling framework that incorporates a spatially weighted cooperative control model. The key innovation in our work is the introduction of the γ-coefficient, which dynamically adjusts the influence of each vehicle based on its relative position in the platoon. This allows for a more flexible representation of vehicle control authority, particularly in heterogeneous traffic environments where vehicle types and communication delays vary.

While previous studies have considered CAV spatial distribution, they have primarily focused on static or simplistic models of vehicle positioning. In contrast, our approach offers a scalable and nonlinear framework that accounts for the spatial heterogeneity of CAVs and their influence on platoon stability across different configurations. The γ-coefficient enables this framework to be applied to a wide range of vehicle compositions, from low to high market penetration of CAVs, providing both theoretical insights and quantitative guidelines for optimizing vehicle deployment strategies in mixed traffic scenarios. By integrating the spatial weighting mechanism with a generalized stability analysis, our model provides a broader and more accurate representation of how vehicle positioning affects platoon dynamics, thus filling a critical gap in the existing literature on mixed vehicle platoons.

This research not only contributes to the academic understanding of platoon stability in mixed traffic but also provides practical implications for the design and deployment of autonomous vehicle fleets. The model can be applied to optimize traffic flow management and platoon formation strategies in real-world mixed traffic scenarios, thus enhancing both the safety and efficiency of autonomous transportation systems.

## 2. Methodology

This section develops a generalized stability framework to quantify the impacts of CAV spatial distribution on mixed traffic platoons composed of Human-Driven Vehicles (HDVs), Automated Vehicles (AVs), and Connected and Automated Vehicles (CAVs). The proposed method integrates two main components: (1) a generalized dynamic model describing heterogeneous vehicle interactions; (2) a spatial-weighted cooperative control law that embeds the position-dependent influence of CAVs. Together, these components enable a unified and scalable approach to analyze the stability of arbitrary mixed platoon configurations.

### 2.1. Generalized dynamic framework for mixed platoons

To generalize the analysis beyond limited configuration cases, we formulate the mixed platoon as a parameterized dynamic system that captures vehicle-type heterogeneity and communication topology. For the i-th vehicle, the longitudinal motion is given by:


$${\dot{\nu }}_{i}(t)=f_{i}(\Delta x_{i},\Delta \nu_{i};\theta_{i})$$ (1)

$$f_{i}(\cdot )=k_{i}\Delta \nu_{i}+\alpha_{i}\Delta x_{i}+\beta_{i}\sum_{j\in N_{i}}\;p_{ij}\left(\nu_{j}-\nu_{i}\right)$$ (2)

where $\Delta x_{i}=x_{i-\text{1}}-x_{i}-s_{\text{0}}$ and $\Delta \nu_{i}=v_{i-\text{1}}-v_{i}$ denote spacing and velocity differences; $\theta_{i}=[k_{i},\;\alpha_{i},\;\beta_{i}]$ are the car-following sensitivity, spacing coefficient, and communication gain; $p_{ij}$ represents the information connectivity between vehicle *i* and *j*, with $p_{ij}=\text{1}$ if data are received and 0 otherwise.

By assembling all *N* vehicles, the system dynamics can be written in compact matrix form:


$$\dot{V}(t)=-K\cdot L(P)\cdot V(t)$$ (3)

where V = ${\left[\nu_{\text{1}},\;\nu_{\text{2}},\;\cdots ,\nu_{N}\;\right]}^{T}$ is the velocity vector, K= diag ($k_{\text{1}},\;k_{\text{2}},\;\cdots ,k_{N}$), and *L*(*P*) is the Laplacian matrix defined by the spatial distribution matrix *P*. The eigenvalue spectrum of ·determines the platoon’s dynamic stability:


$$\text{Re}\left(\lambda_{i}\left(KL(P)\right)\right)<\text{0}\;\forall i$$ (4)

This generalized formulation allows stability evaluation for any arbitrary composition or spatial layout of HDVs, AVs, and CAVs, including but not limited to the 16 specific cases used for scenario demonstration. While traditional cooperative control assumes uniform CAV influence, real-world performance varies with vehicle location. To capture this effect, we introduce a spatial weighting factor *ωi* that dynamically adjusts each vehicle’s control authority based on its distance from the platoon leader. The longitudinal acceleration of the *i*-th CAV is governed by:


$$a_{i}=\omega_{i}\left(k_{i}\Delta \nu_{i}+k_{\text{2}}\Delta x_{i}\right)+\left(\text{1}-\omega_{i}\right)\sum_{j\in N_{i}}\;\beta_{ij}\left(\nu_{j}-\nu_{i}\right)$$ (5)

$$\omega_{i}=\text{exp}\left(\;-\gamma d_{i}\right)\;$$

where 𝑑𝑖 is the normalized distance between vehicle 𝑖 and the leader, and 𝛾 is the spatial sensitivity coefficient controlling the decay rate of CAV influence along the platoon. When 𝛾=0, the model degenerates to uniform cooperative control; when 𝛾>0, vehicles farther from the leader receive proportionally reduced influence from leading CAVs. This spatial-weighted mechanism captures the positional heterogeneity of CAVs, enabling the model to reveal nonlinear relationships between CAV positioning and global string stability—an aspect previously unquantified in the literature (e.g., [11,28]).

In this study, the exponential decay function for the spatial weighting coefficient (γ) was selected to represent the diminishing control influence of vehicles in a platoon as a function of distance from the leader. The choice of an exponential decay function is motivated by its flexibility and widespread use in dynamic systems and multi-agent control, where influence decays sharply over distance. Compared to alternative functions such as linear or inverse distance decay, the exponential form better captures the observed attenuation of control authority, particularly in vehicle platoons where communication delay and dynamic interaction influence the system’s stability. Linear decay may fail to reflect the sharp fall-off in control effectiveness over distance, while inverse distance decay could lead to unrealistic overestimation of the influence of distant vehicles. Empirical evidence from multi-agent systems and cooperative vehicle control further supports the use of exponential decay to model these interactions. Future work could explore the use of other functional forms to evaluate their effect on the platoon dynamics and stability.

### 2.2. String stability analysis and spatially weighted cooperative control

Following the Laplace-domain approach, the transfer function between consecutive vehicles is expressed as:


$$G_{i}(s)=\frac{V_{i}(s)}{V_{i-\text{1}}(s)}=\frac{k_{i}+\alpha_{i}s}{s^{\text{2}}+k_{i}s+\alpha_{i}}$$ (6)

A platoon satisfies string stability if the magnitude of the transfer function does not exceed unity for any frequency [30,31]:


$$|G_{i}(j\omega )|⩽\text{1},\forall \omega >\text{0}$$ (7)

Under the proposed framework, this condition depends not only on vehicle control parameters (𝑘_𝑖_, 𝛼_𝑖_) but also on the eigenvalues of the weighted Laplacian 𝐿(*p*,𝜔_𝑖_), linking stability directly to CAV spatial distribution. This enables systematic evaluation of how local communication and positional weighting jointly affect global dynamic behavior.

The introduction of the spatial weighting coefficient γ is a key innovation in our control model. This coefficient dynamically adjusts the influence each vehicle exerts on the platoon, based on its relative position to the platoon leader. Specifically, γ determines how strongly the control action of each vehicle is influenced by the leader as a function of the distance between vehicles in the platoon. The coefficient γ is crucial for balancing local responsiveness and overall platoon stability.

The coefficient γ serves as a spatial sensitivity factor that modifies the feedback strength between vehicles in the platoon. As the distance between vehicles increases, the influence of the leader on a vehicle decreases exponentially with the value of γ. A larger γ gives higher weight to the control actions of the leader for vehicles closer to it, while a smaller γ distributes the influence more evenly across the platoon, which can help maintain stability in larger formations.

The range of γ (0.3–0.5) is chosen empirically through simulations, which optimize the trade-off between localized control and platoon stability. From a control theory perspective, γ affects the eigenvalues of the platoon’s system dynamics, specifically influencing the system’s stability margin. A larger γ may improve control at the front of the platoon but can destabilize the formation if the platoon becomes too large. A smaller γ, on the other hand, reduces the control influence from the leader and can lead to slower response times. The optimal range of 0.3–0.5 represents a balance where the platoon maintains stability while still reacting promptly to changes in the leader’s behavior.

The value of γ is also influenced by vehicle communication topology and car-following dynamics. In platooning systems, communication delays and control gains are critical factors that affect the responsiveness of each vehicle. As the distance between vehicles increases, communication delays can reduce the effectiveness of the control system, which may require a lower γ to prevent instability. Additionally, in car-following dynamics, the ability of a vehicle to match the leader’s speed and position is directly impacted by γ. A higher γ allows for tighter synchronization, but excessive control can lead to oscillations. Therefore, the choice of γ depends on the desired control precision and the communication structure of the platoon, as well as the vehicle types involved.

In this study, we analyze three types of mixed fleets: AV-HDV, CAV-HDV, and AV-CAV-HDV. To ensure consistency and robustness in the analysis, we propose a unified stability analysis framework that applies to all three types of fleets. This framework accounts for the interaction between AVs, CAVs, and HDVs, ensuring a comprehensive understanding of platoon stability.

The control parameters for AVs, CAVs, and HDVs are defined consistently across all fleet types. The control strategies for AVs and CAVs are based on similar communication mechanisms and feedback control systems. The model parameters, such as vehicle acceleration, communication delays, and control gains, are consistently applied in the analysis for all fleet types.

The coupling relationship between AV and CAV control parameters is explicitly modeled. In mixed fleets, the control inputs of AVs (such as speed and acceleration) and CAVs (such as communication signals and reaction times) are interconnected, with each vehicle’s control authority being influenced by its position in the platoon and its interaction with other vehicles, particularly HDVs. We derive this coupling relationship to more accurately represent the interactions between AVs and CAVs and their effects on platoon stability.

## 3. Stability analysis of mixed vehicle platoon with AV and HDV

**Fig 1:**
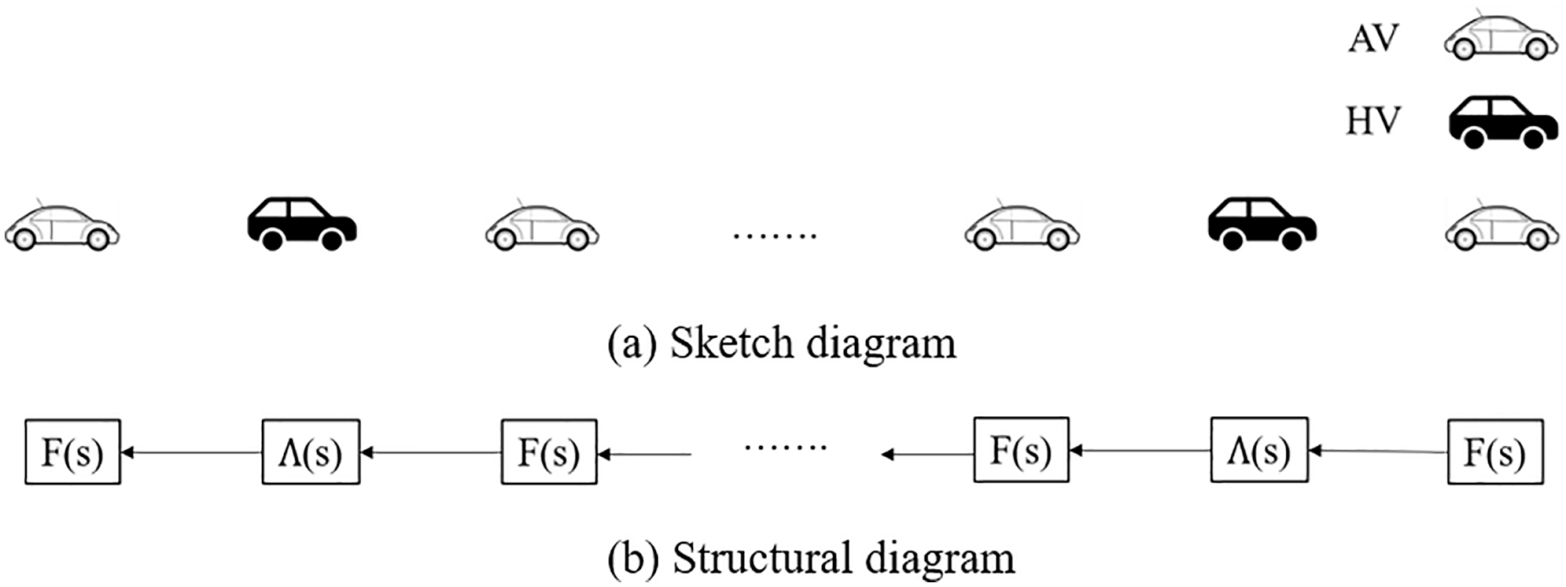
Sketch diagram and structural diagram of mixed vehicle platoon with AV and HDV.

As shown in Fig 1 (a), a mixed vehicle platoon consists solely of AV and HDV on a single lane, in which the HDV behavior is controlled by drivers, and the AV can sense the preceding vehicle states (e.g., the distance between the two vehicles, the preceding vehicle velocity) via fixed sensors such as Lidars. Assuming that all AV are of the same specifications, so are all HDV, and the transfer function of AV is F(s), as well as the transfer function of HDV is Λ(s). To streamline the analysis, it is assumed that the control systems of all AV and CAV are linear. This assumption is well-supported by existing literature, which has extensively utilized linear models to describe vehicle control systems (e.g., Li (2022) [9]; Montanino et al.(2021) [22]), and some commercial AV now use linear controllers. On the other hand, the linear control framework provides a more straightforward representation of the operational characteristics of AV and CAV within heterogeneous traffic scenarios, thereby facilitating a clearer understanding of their system dynamics (18). On the basis of the above assumptions, the platoon in Fig 1 (a) can be converted into the structure diagram form shown as Fig 1 (b).

Under this traffic environment, since there exists no communication behavior between vehicles, neither AV nor HDV can know the type their preceding vehicles. Moreover, AV can only dynamically control their own performances through sensing the preceding vehicle state, hence AV repositioning cannot improve platoon stability. Denote that the market penetration rate of AV is P (0<P≤1), and the number of vehicles in the platoon is n. Thus, the market penetration rate of HDV can be expressed as 1−P, and the transfer function of the whole platoon Q(s) can be expressed as:


$$Q(s)={F(s)}^{nP}{\cdot \Lambda (s)}^{n(\text{1}-P)}$$ (8)

Substitute s=ωj into Eq. 8 to obtain:


$$\text{Q}(\omega j)={F(\omega j)}^{n\text{P}}{\cdot \Lambda (\omega j)}^{n(\text{1}-\text{P})}$$ (9)

Then, the magnitude of Q(s) can be formulated as:


$$\left|\text{Q}(\omega j)\right|=\left|{F(\omega j)}^{n\text{P}}{\cdot \Lambda (\omega j)}^{n(\text{1}-\text{P})}\right|={\left|F(\omega j)\right|}^{n\text{P}}{\cdot \left|\Lambda (\omega j)\right|}^{n(\text{1}-\text{P})}$$ (10)

It is worth noting that, since the behavior of HDV is only controlled by drivers but not control systems (i.e., |Λ(ωj)| is constant), the platoon stability cannot be improved through HDV. Hence, the platoon stability is related to the market penetration rate of AV and the number of vehicles in the platoon n, when the AV and HDV transfer functions are specified, according to Eq. 10.

## 4. Stability analysis of mixed vehicle platoon with CAV and HDV

**Fig 2:**
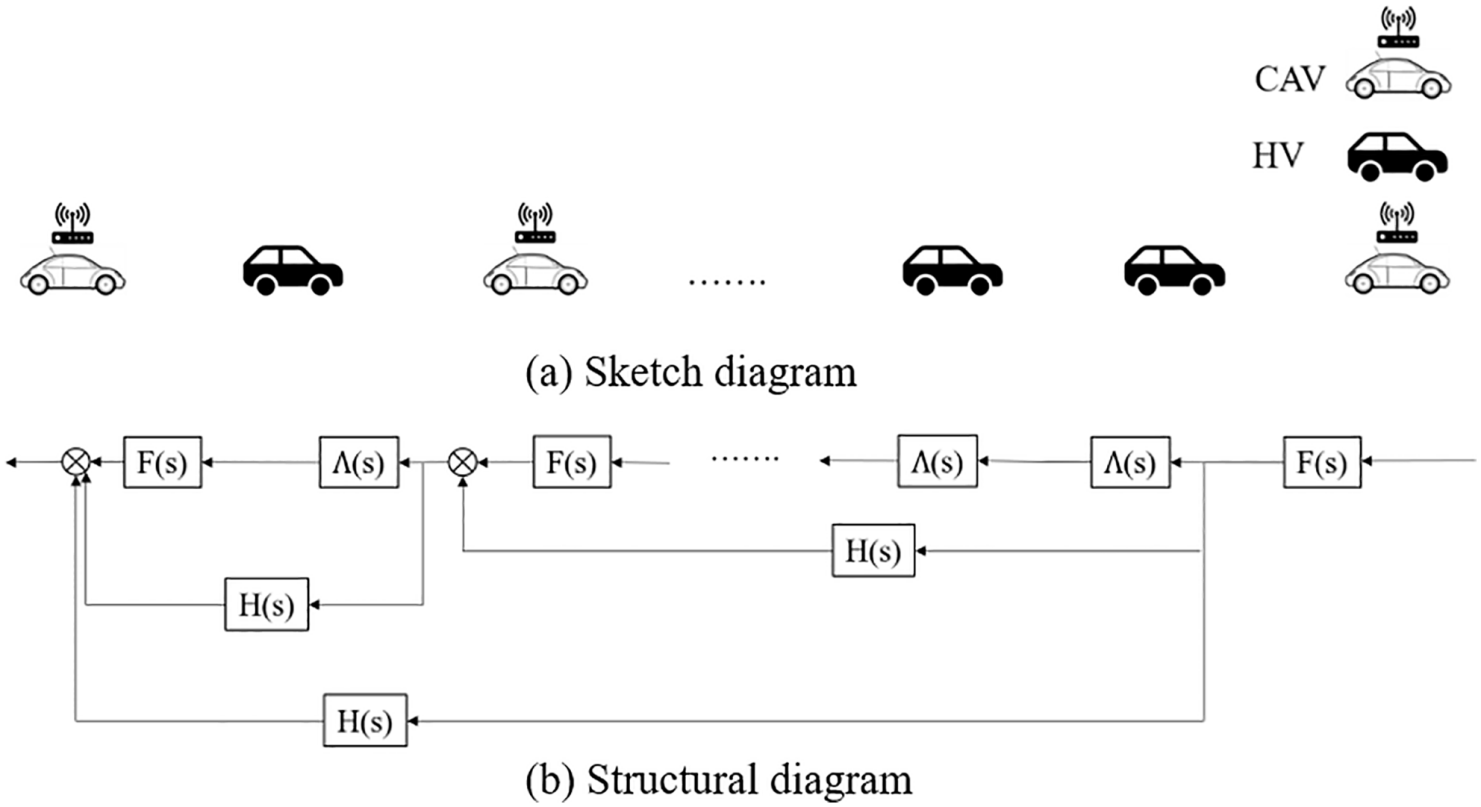
Sketch diagram and structural diagram of mixed vehicle platoon with CAV and HDV.

When only CAV and HDV exist on the road, vehicles can form a platoon as shown in Fig 2 (a). If the transfer function of CAV to the rear vehicle is expressed as F(s), the transfer function of CAV to the network communication is expressed as H(s), and the transfer function of HDV to the rear vehicle is expressed as Λ(s), then Fig 2 (a) can be converted into the CAV and HDV mixed platoon structure diagram as shown in Fig 2 (b). Since CAV can transmit full or part of the information to the rear CAV, the platoon structure diagram will change according to the spatial distribution of CAV in the platoon. Thus, the platoon stability may also be affected by the spatial distribution of CAV. To deeply explore the relationship between CAV spatial distribution and mixed platoon stability, this section analysis the spatial distribution pattern impact on platoon stability from the two aspects: CAV at the front and/or tail of a platoon, as well as CAV internal distribution within a platoon, respectively.

### 4.1. Spatial distribution and stability analysis of CAV at the front/tail of a platoon

**Fig 3:**
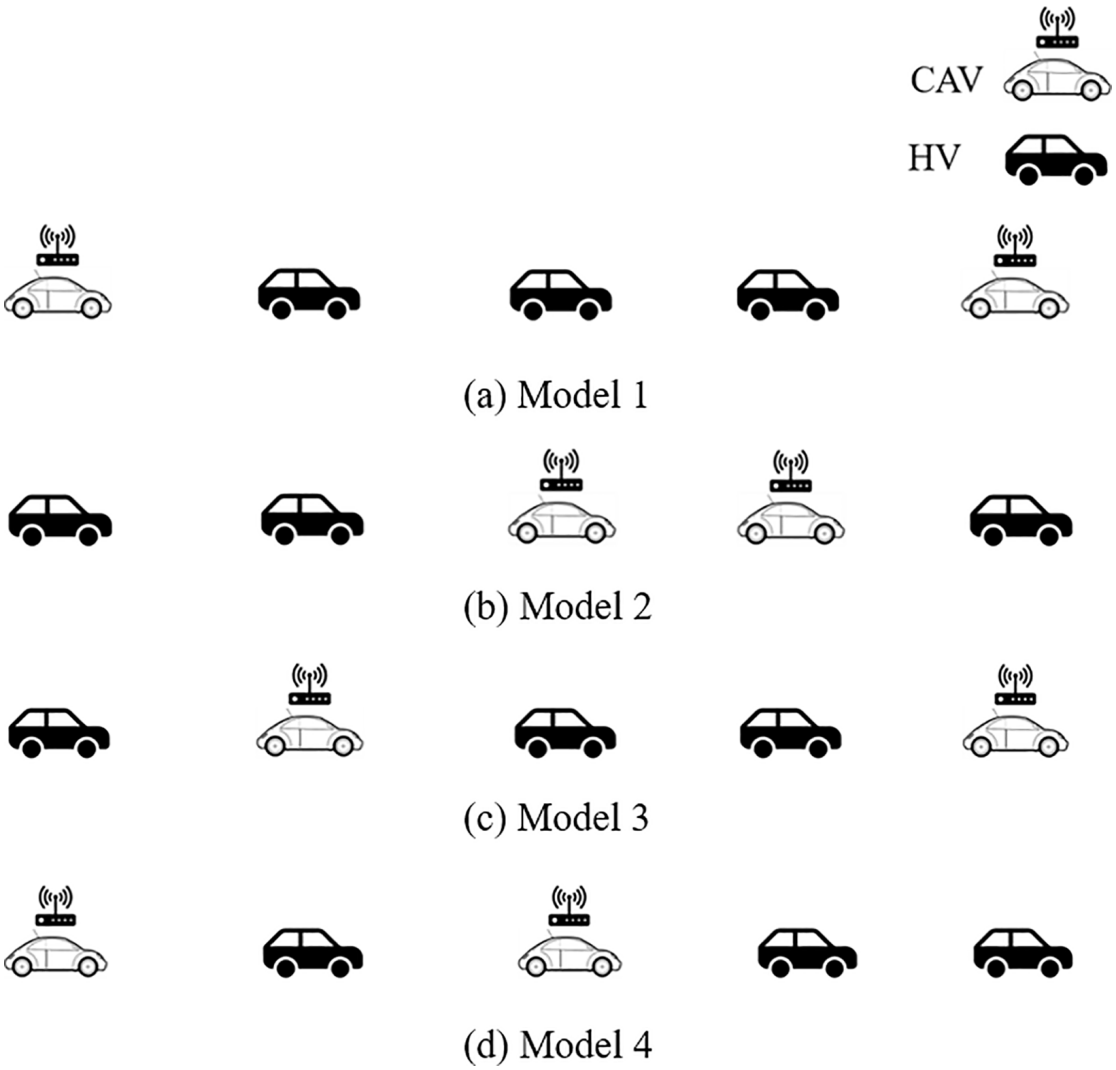
Four typical CAV front and/or tail spatial distribution patterns in a CAV, HDV mixed platoon with 40% CAV market penetrate rate.

Assuming that the vehicle number of the platoon is 5 and the market penetration rate of CAV is 40%. For the CAV front and/or tail distribution as shown in Fig 3, there are four typical distribution forms (labeled as Model 1- Model 4, respectively):

- Model 1: CAV are distributed at the front and tail of the platoon;
- Model 2: CAV are distributed within the platoon;
- Model 3: CAV are distributed at the front and within the platoon;
- Model 4: CAV are distributed at the tail and within the platoon.

**Fig 4:**
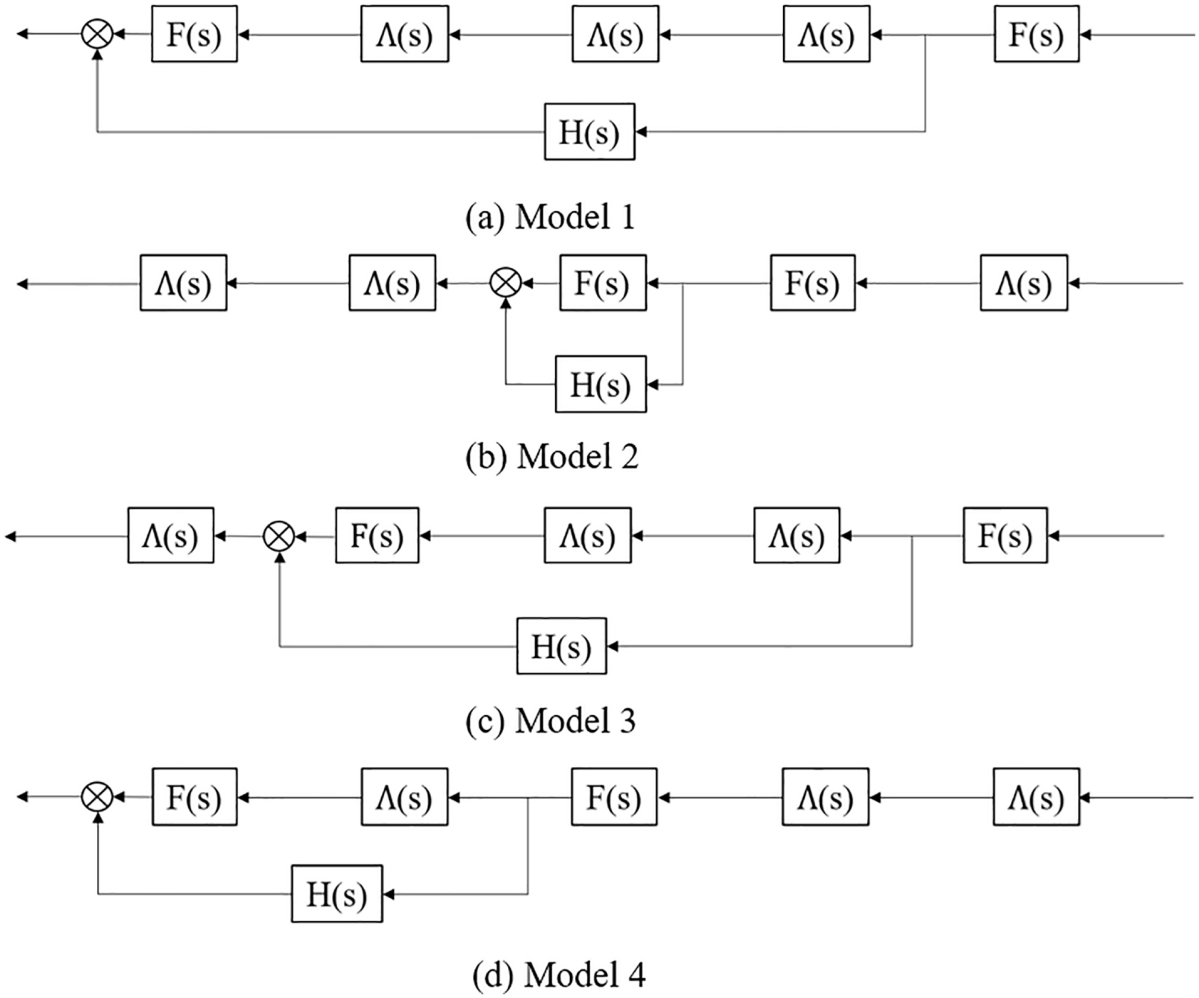
Structure diagram of the four typical CAV front and/or tail spatial distribution patterns in a CAV, HDV mixed platoon.

On the basis of control system characteristics, the above platoon spatial distribution can be converted into the structure diagram form shown in Fig 4. In principle, the transfer function can be calculated:

- Model 1: ${F(s)}^{\text{2}}{\Lambda (s)}^{\text{3}}+F(s)H(s)$
- Model 2: ${F(s)}^{\text{2}}{\Lambda (s)}^{\text{3}}+{\Lambda (s)}^{\text{3}}\;F(s)H(s)$
- Model 3: ${F(s)}^{\text{2}}{\Lambda (s)}^{\text{3}}+\Lambda (s)F(s)H(s)$
- Model 4: ${F(s)}^{\text{2}}{\Lambda (s)}^{\text{3}}+\;{\Lambda (s)}^{\text{2}}\;F(s)H(s)$

It can be found that all four transfer functions contain terms ${\text{F}(\text{s})}^{\text{2}}{\Lambda (\text{s})}^{\text{3}}$, which represents the platoon scenario where no network communication is considered. For the purpose of exploring the effect of CAV spatial distribution on the mixed platoon stability, the differences in the transfer function need to be further analyzed. Taking the transfer function of Model 1 as an example. Substitute s=ωj into Model 1 transfer function, and the stability condition of Model 1 can be expressed as follows:


$${\left|F(\omega j\right)}^{\text{2}}{\Lambda (\omega j)}^{\text{3}}+F(\omega j)H(\omega j)|<\text{1}$$ (11)

To bring more insights into Eq. 11, Eq. 12 can be obtained:


$${\left|F(\omega j\right)}^{\text{2}}{\Lambda (\omega j)}^{\text{3}}+F(\omega j)H(\omega j)\left|\leq {\left|F(\omega j\right)}^{\text{2}}{\Lambda (\omega j)}^{\text{3}}\right|+|F(\omega j)H(\omega j)|$$ (12)

Therefore, if Equation 13 is true, the Eq. 11 must be true:


$$\left|{F(\omega j)}^{\text{2}}{\Lambda (\omega j)}^{\text{3}}\right|+|F(\omega j)H(\omega j)|<\text{1}$$ (13)

where ${\left|\text{F}(\omega \text{j}\right)}^{\text{2}}{\Lambda (\omega \text{j})|}^{\text{3}}$ can be convert into ${\left|\text{F}(\omega \text{j})\right|}^{\text{2}}{\left|\Lambda (\omega \text{j})\right|}^{\text{3}}$. Hence, the value of $\left|{\text{F}(\omega \text{j})}^{\text{2}}{\Lambda (\omega \text{j})}^{\text{3}}\right|$, which is the same in the four models, is only related to the value of |Λ(ωj)| and |F(ωj)|. Moreover, the differences in the four transfer function can be written as:

- Model 1: |F(ωj)H(ωj)|=|F(ωj)|·|H(ωj)|
- Model 2: $\left|{\Lambda (\omega j)}^{\text{3}}\;F(\omega j)H(\omega j)\right|={\left|\Lambda (\omega j)\right|}^{\text{3}}\cdot \left|F(\omega j)\right|\cdot |H(\omega j)|$
- Model 3: |Λ(ωj)F(ωj)H(ωj)|=|Λ(ωj)|·|F(ωj)|·|H(ωj)|
- Model 4: $\left|{\Lambda (\omega j)}^{\text{2}}F(\omega j)H(\omega j)\right|={\left|\Lambda (\omega j)\right|}^{\text{2}}\cdot \left|F(\omega j)\right|\cdot |H(\omega j)|$

Note that the value of |Λ(ωj)| depends on HDV, which is usually over 1. It can be found that, the differences of platoon stability between models are depend on the order of |Λ(ωj)|. Hence, Model 1, where CAV are distributed at the front and tail of the platoon, can better ensure the platoon stability. Under Model 1 conditions, the leading CAV can transmit external interference information (e.g., speed and/or spacing fluctuations) directly to the rear CAV. Consequently, the entire platoon can promptly address external disturbances in a cohesive manner. In general, in order to improve platoon stability, if the market penetration rate of CAV is low, the leading vehicle should ensure to be CAV; when the market penetration rate of CAV is relatively increased, part of the CAV can be distributed at the tail of the platoon on the premise that the leading vehicle is CAV.

### 4.2. Spatial distribution and stability analysis of CAV within a platoon

**Fig 5:**
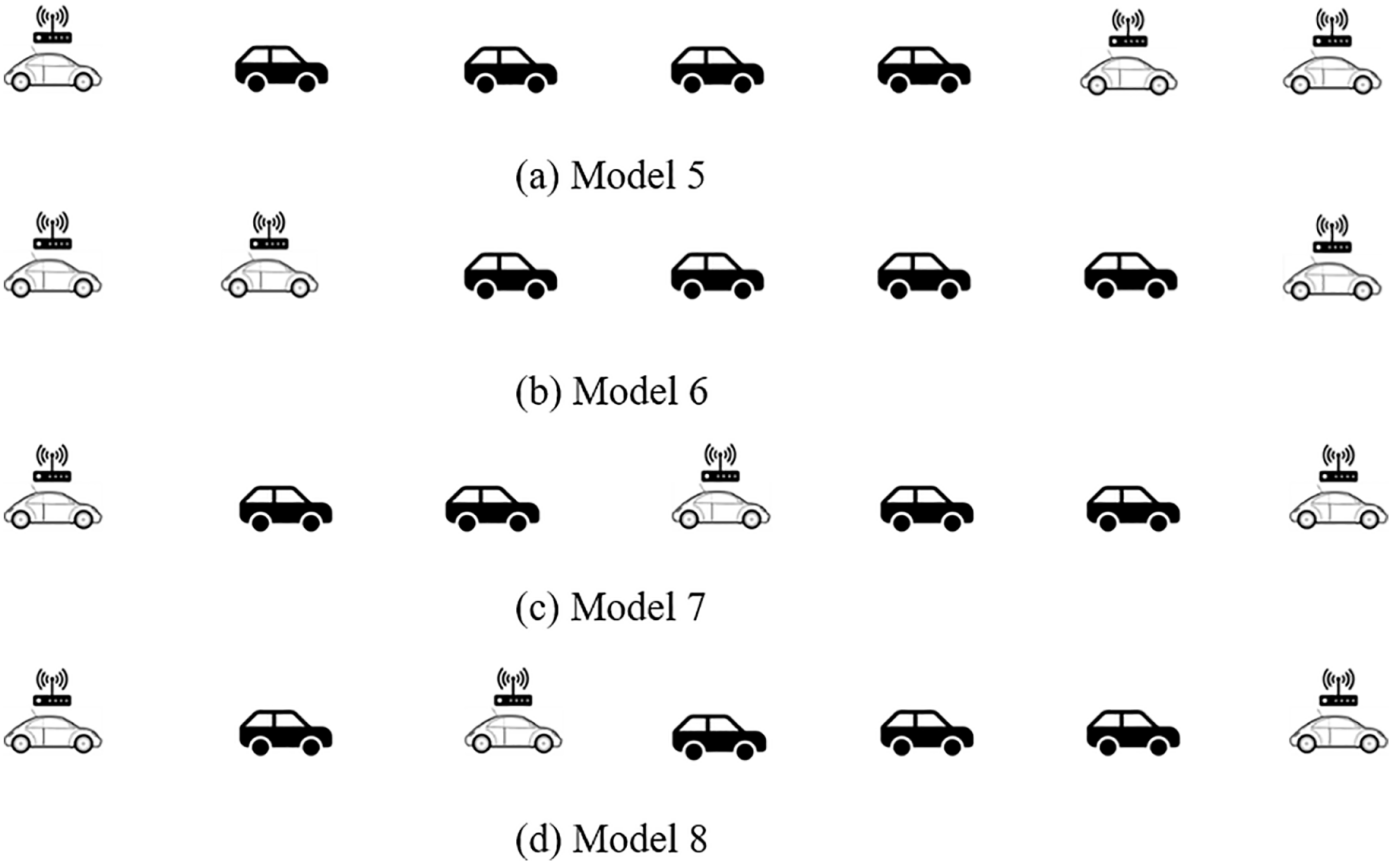
Four typical CAV within a CAV, HDV mixed platoon spatial distribution patterns with 40% CAV market penetrate rate.

Further, when the vehicle number of platoon increases or the market penetration rate of CAV is further improved, the spatial distribution and stability analysis of CAV within a platoon need to be explored. Assuming that the vehicle number of the platoon increases to 7, the market penetration rate of CAV is still 40%, and there have existed CAV distributed at the front and tail of the platoon. For the CAV within a platoon distribution, Fig 5 indicates four typical distribution forms (labeled as Model 5 – Model 8, respectively):

- Model 5: CAV are only distributed at the front and tail of the platoon and mainly at the front;
- Model 6: CAV are only distributed at the front and tail of the platoon and mainly at the tail;
- Model 7: CAV are distributed at the front and tail of the platoon and CAV are uniformly distributed in the platoon;
- Model 8: CAV are distributed at the front and tail of the platoon and CAV are randomly distributed in the platoon.

**Fig 6:**
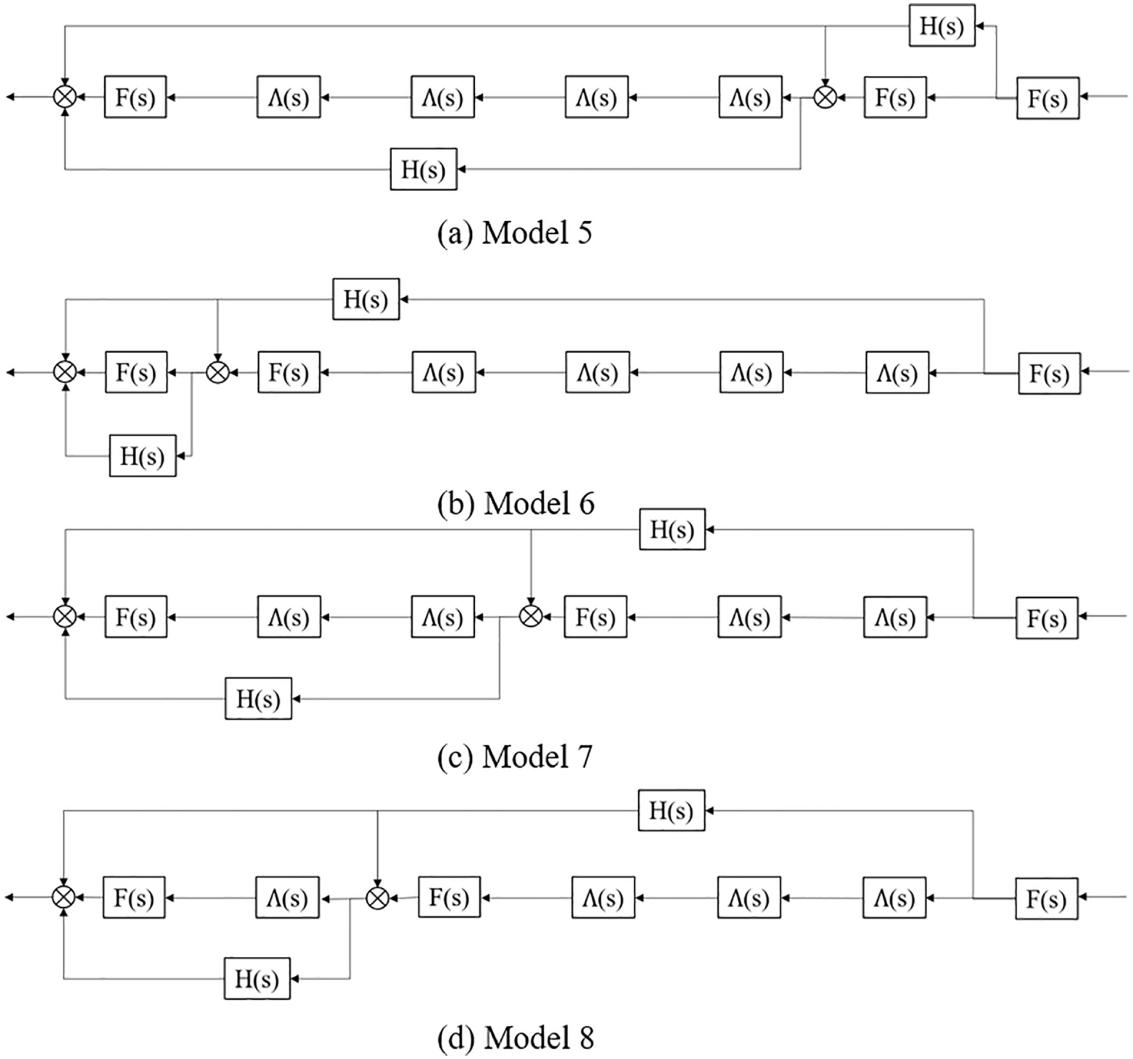
Structure diagram of the four typical CAV within a CAV, HDV mixed platoon spatial distribution patterns.

On the basis of control system characteristics, the above platoon spatial distribution can be converted into the structure diagram form shown in Fig 6. Therefore, the transfer functions of platoons Model 5 to Model 8 can be calculated as:

- Model 5: ${F(s)}^{\text{3}}{\Lambda (s)}^{\text{4}}+F(s){H(s)}^{\text{2}}+F(s)H(s)+{F(s)}^{\text{2}}H(s){\Lambda (s)}^{\text{4}}+{F(s)}^{\text{2}}H(s)$;
- Model 6: ${F(s)}^{\text{3}}{\Lambda (s)}^{\text{4}}+F(s){H(s)}^{\text{2}}+F(s)H(s)+{F(s)}^{\text{2}}H(s){\Lambda (s)}^{\text{4}}+{F(s)}^{\text{2}}H(s)$;
- Model 7: ${F(s)}^{\text{3}}{\Lambda (s)}^{\text{4}}+F(s){H(s)}^{\text{2}}+F(s)H(s)+\text{2}{F(s)}^{\text{2}}H(s){\Lambda (s)}^{\text{2}}$;
- Model 8: ${F(s)}^{\text{3}}{\Lambda (s)}^{\text{4}}+F(s){H(s)}^{\text{2}}+F(s)H(s)+{F(s)}^{\text{2}}H(s){\Lambda (s)}^{\text{3}}+{F(s)}^{\text{2}}H(s)\Lambda (s)$.

Obviously, all the four spatial distribution patterns contain ${F(s)}^{\text{3}}{\Lambda (s)}^{\text{4}}+F(s){H(s)}^{\text{2}}+F(s)H(s)$*.* In addition, the transfer function of Model 5 and the transfer function of Model 6 are the same, which means if CAV are distributed at the front and tail of the platoon, adding more CAV at either the front or tail does not affect the platoon stability. Substituting s=ωj into Model 6 transfer function as an illustrative example, the stability condition of Model 6 can be formulated as:


$$|{F(\omega j)}^{\text{3}}{\Lambda (\omega j)}^{\text{4}}+F(\omega j){H(\omega j)}^{\text{2}}+F(\omega j)H(\omega j)+{F(\omega j)}^{\text{2}}H(\omega j){\Lambda (\omega j)}^{\text{4}}+{F(\omega j)}^{\text{2}}H(\omega j)|<\text{1}$$ (14)

Since:


$$\begin{array}{cc} & |{F(\omega j)}^{\text{3}}{\Lambda (\omega j)}^{\text{4}}+F(\omega j){H(\omega j)}^{\text{2}}+F(\omega j)H(\omega j)+{F(\omega j)}^{\text{2}}H(\omega j){\Lambda (\omega j)}^{\text{4}}\\ & +\;{F(\omega j)}^{\text{2}}H(\omega j)|\leq |{F(\omega j)}^{\text{3}}{\Lambda (\omega j)}^{\text{4}}+F(\omega j){H(\omega j)}^{\text{2}}\\ & +\;F(\omega j)H(\omega j)|+\left|{F(\omega j)}^{\text{2}}H(\omega j){\Lambda (\omega j)}^{\text{4}}\right|+|{F(\omega j)}^{\text{2}}H(\omega j)| \end{array}$$ (15)

when inequation (16) holds true, inequation (14) is necessarily satisfied.


$$\begin{array}{cc} & \left|{F(\omega j)}^{\text{3}}{\Lambda (\omega j)}^{\text{4}}+F(\omega j){H(\omega j)}^{\text{2}}+F(\omega j)H(\omega j)\right|\\ & +\left|{F(\omega j)}^{\text{2}}H(\omega j){\Lambda (\omega j)}^{\text{4}}\right|+|{F(\omega j)}^{\text{2}}H(\omega j)|<\text{1} \end{array}$$ (16)

Additionally, when ignoring the term $\left|{F(\omega j)}^{\text{3}}{\Lambda (\omega j)}^{\text{4}}+F(\omega j){H(\omega j)}^{\text{2}}+F(\omega j)H(\omega j)\right|$, the differences between models can be described as:

- Model 5: ${\left|F(\omega j)\right|}^{\text{2}}\cdot \left|H(\omega j)\right|\cdot {\left|\Lambda (\omega j)\right|}^{\text{4}}+{\left|F(\omega j)\right|}^{\text{2}}\cdot |H(\omega j)|$;
- Model 6: ${\left|F(\omega j)\right|}^{\text{2}}\cdot \left|H(\omega j)\right|\cdot {\left|\Lambda (\omega j)\right|}^{\text{4}}+{\left|F(\omega j)\right|}^{\text{2}}\cdot |H(\omega j)|$;
- Model 7: 2${\left|F(\omega j)\right|}^{\text{2}}\cdot |H(\omega j)|\cdot {\left|\Lambda (\omega j)\right|}^{\text{2}}$;
- Model 8: ${\left|F(\omega j)\right|}^{\text{2}}\cdot \left|H(\omega j)\right|\cdot {\left|\Lambda (s)\right|}^{\text{3}}+{\left|F(\omega j)\right|}^{\text{2}}\cdot |H(\omega j)|\cdot |\Lambda (\omega j)|$.

It can be seen that the differences in each model are mainly determined by HDV. Therefore, when the instability of HDV is high, the CAV spatial arrangement of Model 7 can better improve the stability of mixed platoon. Namely, when CAV market penetration rate or the vehicle number of a platoon increases gradually, the uniform distribution of CAV is conducive to improving the stability of the mixed platoon on the basis of satisfying that both the leading and the rear vehicle in the platoon are CAV.

## 5. Stability analysis of mixed vehicle platoon with AV, CAV and HDV

**Fig 7:**
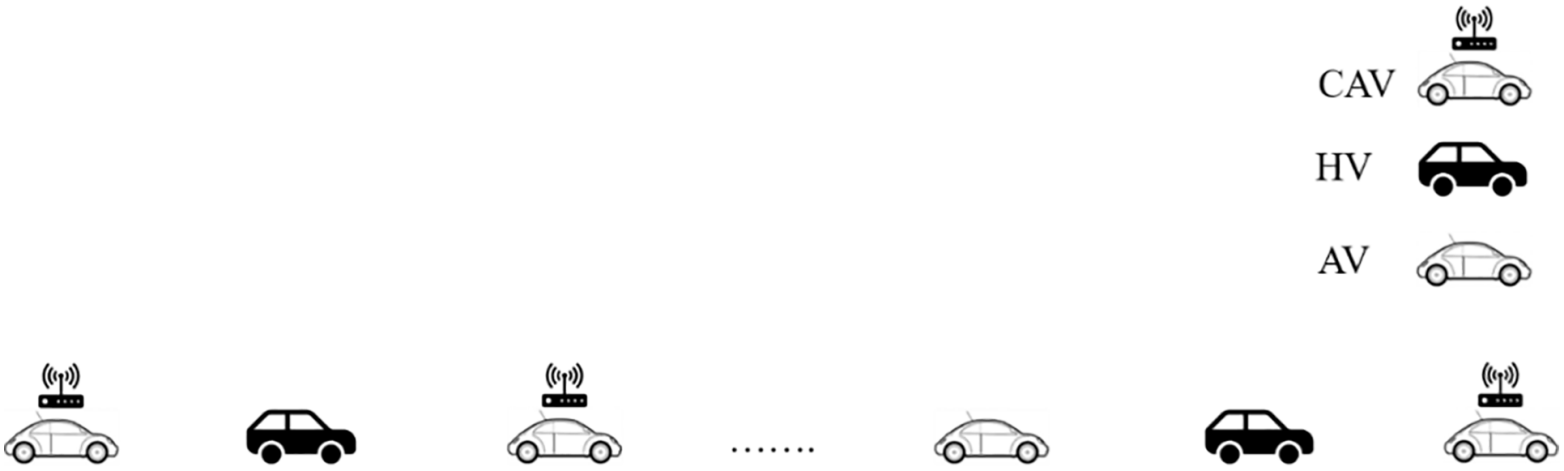
Sketch diagram of mixed vehicle platoon with AV, CAV and HDV.

As shown in Fig 7, the sketch diagram of mixed vehicles platoon with AV, CAV, and HDV is deployed. Likewise, AV and HDV cannot communicate with each other.

**Fig 8:**
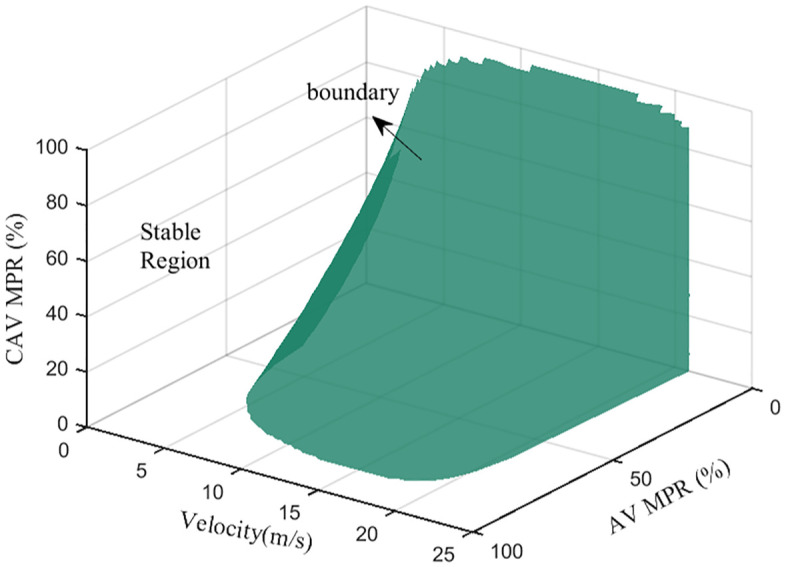
CAV, AV, HDV mixed platoon stable/unstable region.

According to Wilson and Ward’s research [31], if the spatial distribution of vehicles in the mixed platoon is not taken into consideration, the platoon stable condition can be described as:


$${\text{P}}_{\text{1}}({ff}_{AV})+{\text{P}}_{\text{2}}\left({ff}_{CAV}\right)+\left(\text{1}-{\text{P}}_{\text{1}}-{\text{P}}_{\text{2}}\right)\left({ff}_{HV}\right)<\text{0}$$ (17)

where, ${\text{P}}_{\text{1}}$ represents the market penetration rate of AV, and ${\text{P}}_{\text{2}}$ represents the market penetration rate of CAV. In addition:


$${ff}_{AV}=\frac{\text{1}}{\text{2}}{\left(\overset{v}{\underset{AV}{f}}\right)}^{\text{2}}-\overset{\Delta v}{\underset{AV}{f}}\overset{v}{\underset{AV}{f}}-\overset{h}{\underset{AV}{f}}$$ (18)

$${ff}_{CAV}=\frac{\text{1}}{\text{2}}{\left(\overset{v}{\underset{CAV}{f}}\right)}^{\text{2}}-\overset{\Delta v}{\underset{CAV}{f}}\overset{v}{\underset{CAV}{f}}-\overset{h}{\underset{CAV}{f}}$$ (19)

$${ff}_{HV}=\frac{\text{1}}{\text{2}}{\left(\overset{v}{\underset{HV}{f}}\right)}^{\text{2}}-\overset{\Delta v}{\underset{HV}{f}}\overset{v}{\underset{HV}{f}}-\overset{h}{\underset{HV}{f}}$$ (20)

where $\overset{\text{v}}{\underset{\text{M}}{\text{f}}}$ represents the partial differential of the following model expression of vehicle M with respect to the velocity under the platoon equilibrium state; $\overset{\Delta \text{v}}{\underset{\text{M}}{\text{f}}}$ stands for the partial differential of the following model expression of vehicle M with respect to the speed difference Δv between the preceding vehicle and vehicle M under the platoon equilibrium state; $\overset{\text{h}}{\underset{\text{M}}{\text{f}}}$ is the partial differential of the following model expression of vehicle M with respect to the distance h between the preceding vehicle and vehicle M under the platoon equilibrium state. Based on previous research [32], when the free flow speed is 120 km/h and the ideal vehicle control time interval is set to 0.8s, the stability of the mixed platoon with different AV and CAV market penetration rates is shown in Fig 8. In this context, the performance of following HDV is represented by Intelligent Driver Model (IDM) model, the performance of following AV is represented by Adaptive Cruise Control (ACC) model, and the performance of following CAV is represented by Cooperative Adaptive Cruise Control (CACC) model. Consequently, with the increase of AV and CAV market penetration rates, the stability of a mixed platoon will gradually be improved. Based on Section 3, this section will further verify the influence of CAV spatial distribution on the stability of CAV, AV and HDV mixed platoon.

### 5.1. Spatial distribution and stability analysis of CAV at the front/tail of a platoon

**Fig 9:**
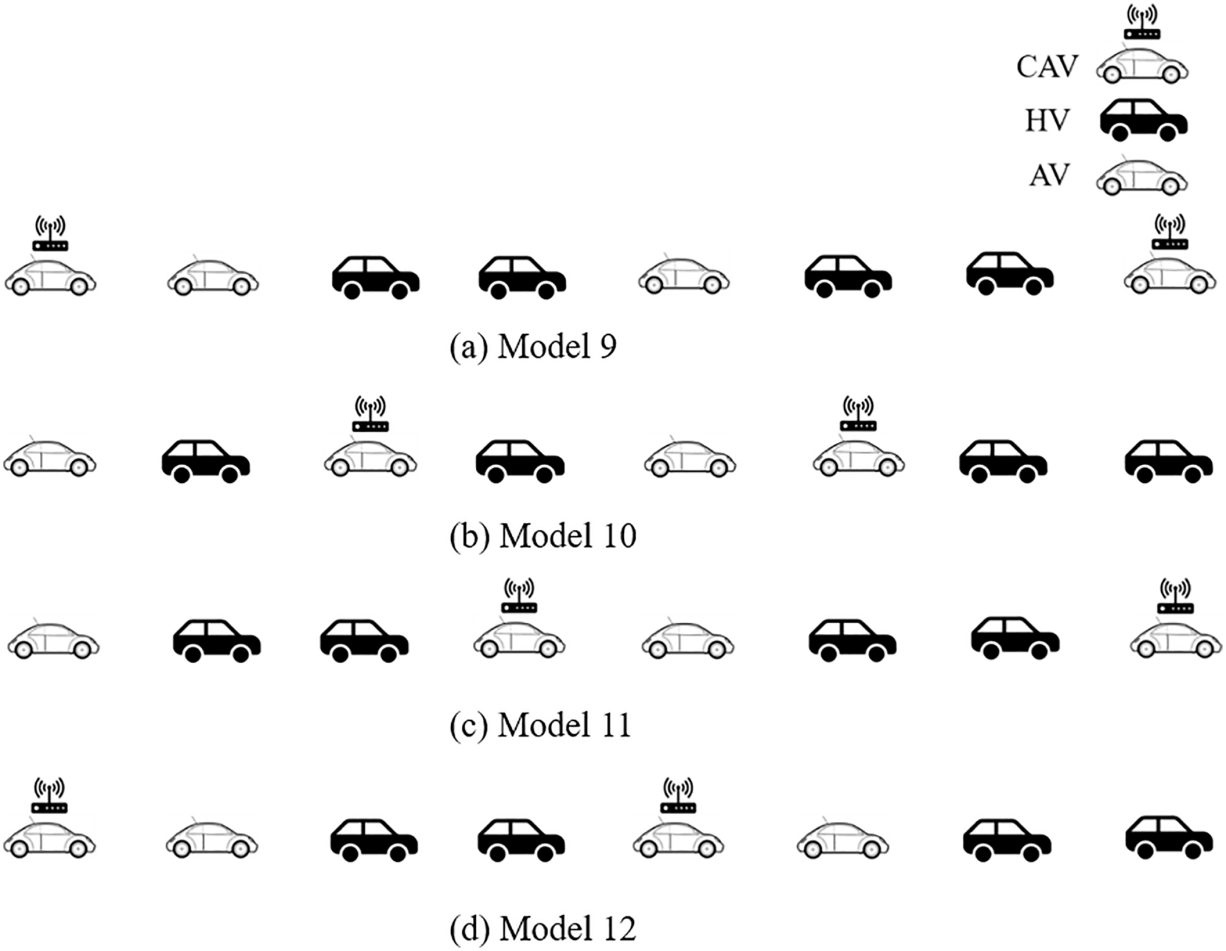
Four typical CAV front and/or tail spatial distribution patterns with 25% CAV and 25% AV market penetrate rates in a AV, CAV, HDV mixed platoon.

Assuming that there exists an 8-vehicle platoon where the market penetration rates of CAV and AV are both 25%, and AV and HDV are randomly distributed in this platoon. Without losing generality, for the front and tail distribution of CAV in the platoon as showcased in Fig 9, there are four typical distribution forms (labeled as Model 5 – Model 8, respectively):

- Model 9: CAV are distributed at the front and tail of the platoon;
- Model 10: CAV are distributed within the platoon;
- Model 11: CAV are distributed at the front and within the platoon;
- Model 12: CAV are distributed at the tail and within the platoon.

**Fig 10:**
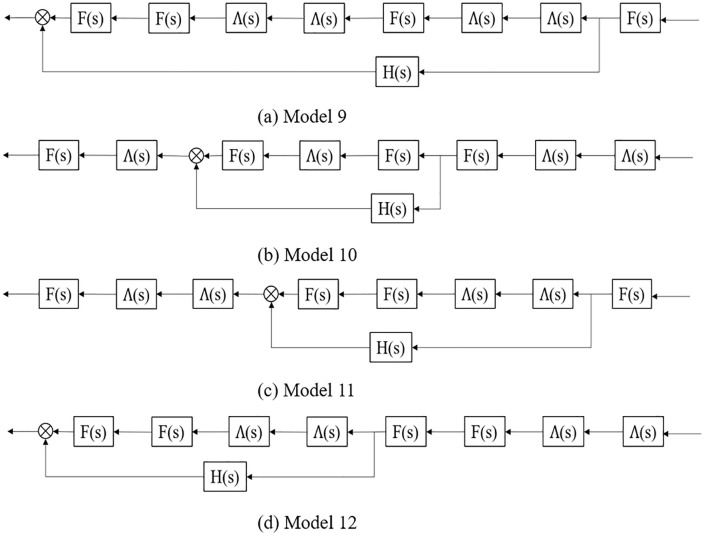
Structure diagram of the four typical CAV front and/or tail spatial distribution patterns with 25% CAV and 25% AV market penetrate rates in a AV, CAV, HDV mixed platoon.

In Fig 10, the above platoon spatial distribution can be converted into the structure diagram form. Then, the transfer functions of platoons Model 9 to Model 12 can be calculated as:

- Model 9: ${F(s)}^{\text{4}}{\Lambda (s)}^{\text{4}}+F(s)H(s)$;
- Model 10: ${F(s)}^{\text{4}}{\Lambda (s)}^{\text{4}}+{\Lambda (s)}^{\text{3}}\;F(s)H(s)$;
- Model 11: ${F(s)}^{\text{4}}{\Lambda (s)}^{\text{4}}+{\Lambda (s)}^{\text{2}}\;{F(s)}^{\text{2}}H(s)$;
- Model 12: ${F(s)}^{\text{4}}{\Lambda (s)}^{\text{4}}+\;{\Lambda (s)}^{\text{2}}\;{F(s)}^{\text{2}}H(s)$.

Taking the transfer function of Model 10 as an example. As before, substitute s=ωj into Model 10 transfer function, and the stability condition of Model 10 can be expressed as follows:


$${\left|F(\omega j\right)}^{\text{4}}{\Lambda (\omega j)}^{\text{4}}+{\Lambda (\omega j)}^{\text{3}}F(\omega j)H(\omega j)|<\text{1}$$ (21)

Besides:


$${\left|F(\omega j\right)}^{\text{4}}{\Lambda (\omega j)}^{\text{4}}+{\Lambda (\omega j)}^{\text{3}}F(\omega j)H(\omega j)\left|\leq {\left|F(\omega j\right)}^{\text{4}}{\Lambda (\omega j)}^{\text{4}}\right|+|{\Lambda (\omega j)}^{\text{3}}F(\omega j)H(\omega j)|$$ (22)

when inequation (23) can be satisfied, inequation (21) is necessarily satisfied.


$$\left|{F(\omega j)}^{\text{4}}{\Lambda (\omega j)}^{\text{4}}\right|+|{\Lambda (\omega j)}^{\text{3}}F(\omega j)H(\omega j)|<\text{1}$$ (23)

When ignoring the term $\left|{\text{F}(\omega \text{j})}^{\text{4}}{\Lambda (\omega \text{j})}^{\text{4}}\right|$, the differences between models can be described as:

- Model 9: |F(ωj)H(ωj)|=|F(ωj)|·|H(ωj)|;
- Model 10: $\left|{\Lambda (\omega j)}^{\text{3}}\;F(\omega j)H(\omega j)\right|={\left|\Lambda (\omega j)\right|}^{\text{3}}\cdot \left|F(\omega j)\right|\cdot |H(\omega j)|$;
- Model 11: |Λ(ωj)F(ωj)H(ωj)|=|Λ(ωj)|·|F(ωj)|·|H(ωj)|;
- Model 12: $\left|{\Lambda (\omega j)}^{\text{2}}F(\omega j)H(\omega j)\right|={\left|\Lambda (\omega j)\right|}^{\text{2}}\cdot \left|F(\omega j)\right|\cdot |H(\omega j)|$.

As a result, Model 9, where CAV are distributed at the front and tail of the platoon, can better ensure the platoon stability. It follows that, in order to improve platoon stability, if the market penetration rate of CAV is low, the leading vehicle should ensure to be CAV; when the market penetration rate of CAV is relatively increased, part of the CAV can be distributed at the tail of the platoon on the premise that the leading vehicle is CAV.

### 5.2. Spatial distribution and stability analysis of CAV within a platoon

**Fig 11:**
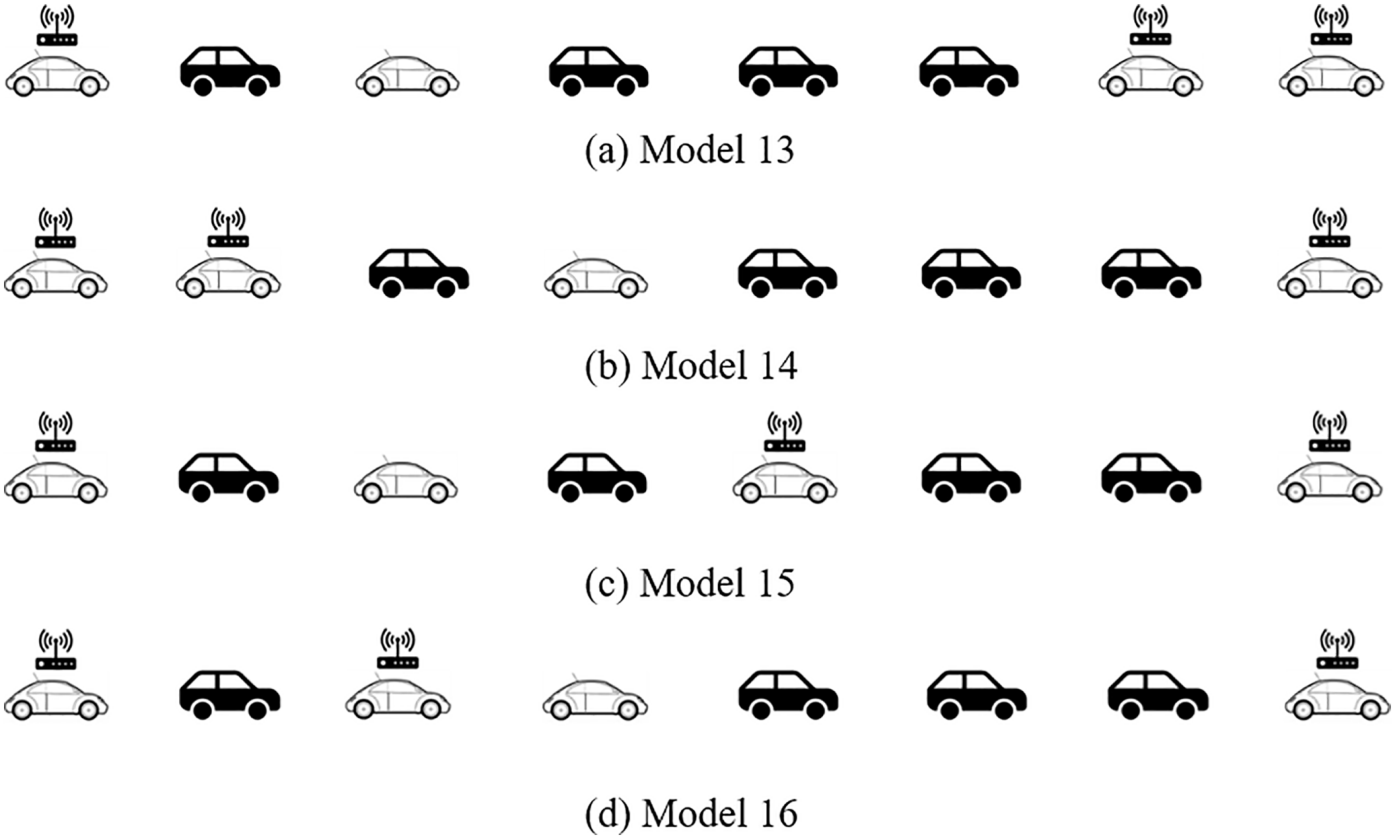
Four typical CAV within platoon spatial distribution patterns with 37.5% CAV and 12.5% AV market penetrate rates in a AV, CAV, HDV mixed platoon.

Assuming that the market penetration rate of CAV increases to 37.5%, and the market penetration rate of AV decreases to 12.5%. For consistence, for the CAV within a platoon distribution as shown in Fig 11, there are four typical distribution forms (labeled as Model 13 – Model 16, respectively):

- Model 13: CAV are only distributed at the front and tail of the platoon and mainly at the front;
- Model 14: CAV are only distributed at the front and tail of the platoon and mainly at the tail;
- Model 15: CAV are distributed at the front and tail of the platoon and CAV are uniformly distributed in the platoon;
- Model 16: CAV are distributed at the front and tail of the platoon and CAV are randomly distributed in the platoon.

**Fig 12:**
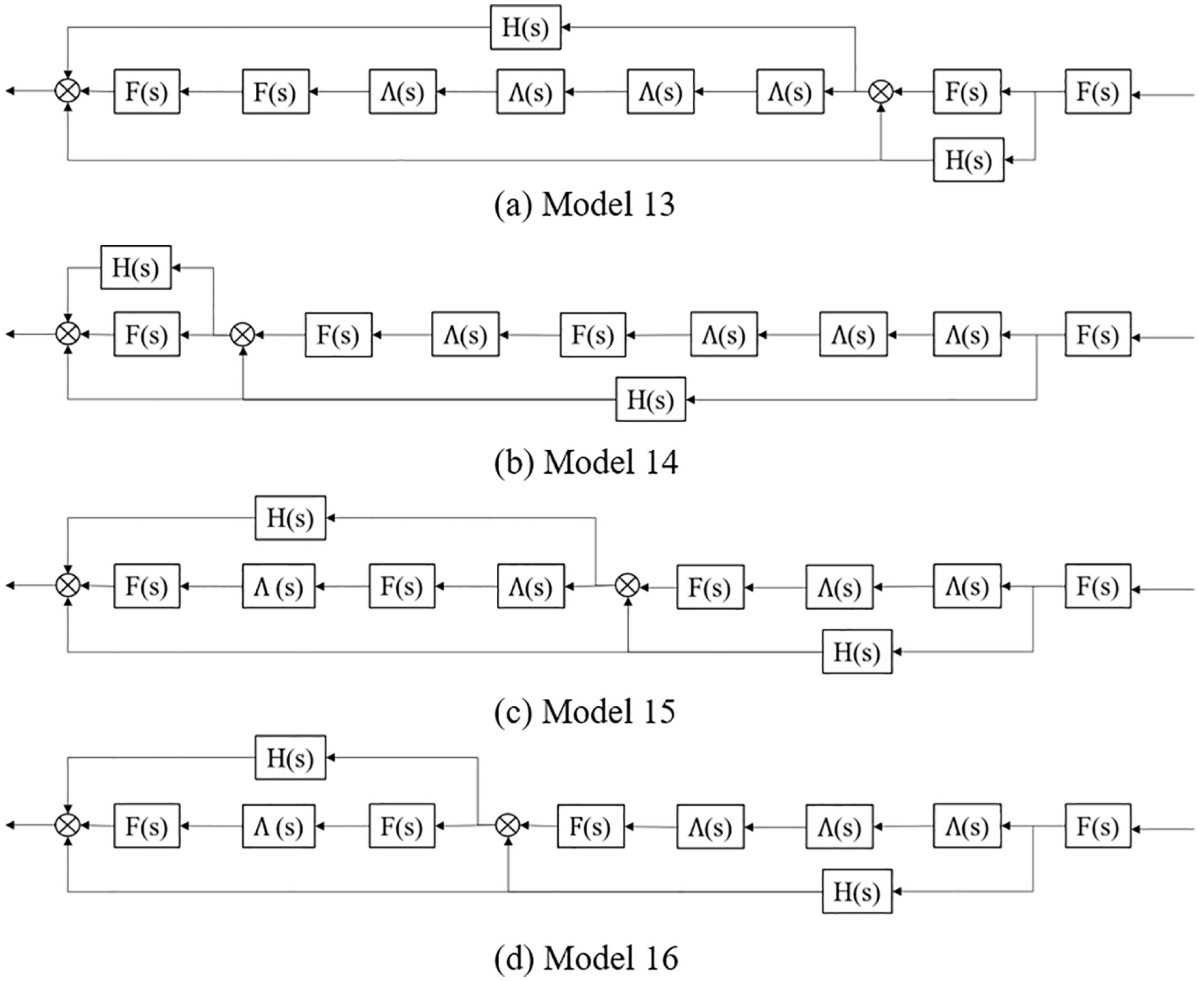
Structure diagram of the four typical CAV within platoon spatial distribution patterns with 37.5% CAV and 12.5% AV market penetrate rates in a AV, CAV, HDV mixed platoon.

The above platoon spatial distribution can be converted into the structure diagram form illustrated in Fig 12, thereof the transfer functions are calculated as:

- Model 13: ${F(s)}^{\text{4}}{\Lambda (s)}^{\text{4}}+F(s)H(s)+F(s){H(s)}^{\text{2}}+{F(s)}^{\text{2}}H(s)+{\Lambda (s)}^{\text{4}}\;{F(s)}^{\text{3}}H(s)$;
- Model 14: ${F(s)}^{\text{4}}{\Lambda (s)}^{\text{4}}+F(s)H(s)+F(s){H(s)}^{\text{2}}+{F(s)}^{\text{2}}H(s)+{\Lambda (s)}^{\text{4}}\;{F(s)}^{\text{3}}H(s)$;
- Model 15: ${F(s)}^{\text{4}}{\Lambda (s)}^{\text{4}}+F(s)H(s)+F(s){H(s)}^{\text{2}}+{\Lambda (s)}^{\text{2}}\;{F(s)}^{\text{2}}H(s)+{\Lambda (s)}^{\text{2}}\;{F(s)}^{\text{3}}H(s)$;
- Model 16: ${F(s)}^{\text{4}}{\Lambda (s)}^{\text{4}}+F(s)H(s)+F(s){H(s)}^{\text{2}}+{\Lambda (s)}^{\text{3}}\;{F(s)}^{\text{2}}H(s)+\Lambda (s){F(s)}^{\text{3}}H(s)$.

On this basis, when HDV hold high instability, Model 15 is more stable than others.

## 6. Numerical simulation and safety analysis

The proposed framework was implemented in MATLAB/Simulink using a mixed platoon consisting of 10 vehicles with varying proportions and spatial distributions of HDVs, AVs, and CAVs. The vehicle dynamics follow the generalized stability model described in Section 2.1, and each CAV applies the spatial-weighted cooperative control law with sensitivity coefficient 𝛾∈[0,1.0]. In simulation, the maximum acceleration of all vehicles is 4 m/s^2^, the maximum deceleration is 5 m/s^2^, the simulation time is 100s, all vehicles are in equilibrium state at the initial moment, and the leading vehicle in all platoons suffer from the acceleration disturbance demonstrated as follows:


$${\text{a}}_{\text{n}}(\text{t})=\left\{ \begin{array}{c} @l\text{0}\text{ m}/{\text{s}}^{\text{2}}\;t\leq \text{10s}\\ -\text{5}\times \text{sgn}(\text{sin}(\text{0}.\text{005}*t))\text{ m}/{\text{s}}^{\text{2}}\text{ 10s}<t\leq \text{60s}\\ \text{0}\text{ m}/{\text{s}}^{\text{2}}\text{ 60s}<t\leq \text{100s} \end{array}\right.\;$$ (24)

To ensure the robustness of the results, multiple simulation runs were conducted for each scenario with different random seeds and initial conditions. The key performance metrics, such as distance gap and velocity fluctuations, were computed for each simulation run. The variance across these runs was analyzed to assess the variability of the system’s response. In addition to the mean values, the results are presented with corresponding standard deviations, and confidence intervals (at a 95% confidence level) are reported for the most critical outcomes. Significance tests, such as ANOVA or t-tests, were performed where appropriate to assess the statistical robustness of the findings and ensure the validity of the results under varying initial conditions.

**Fig 13:**
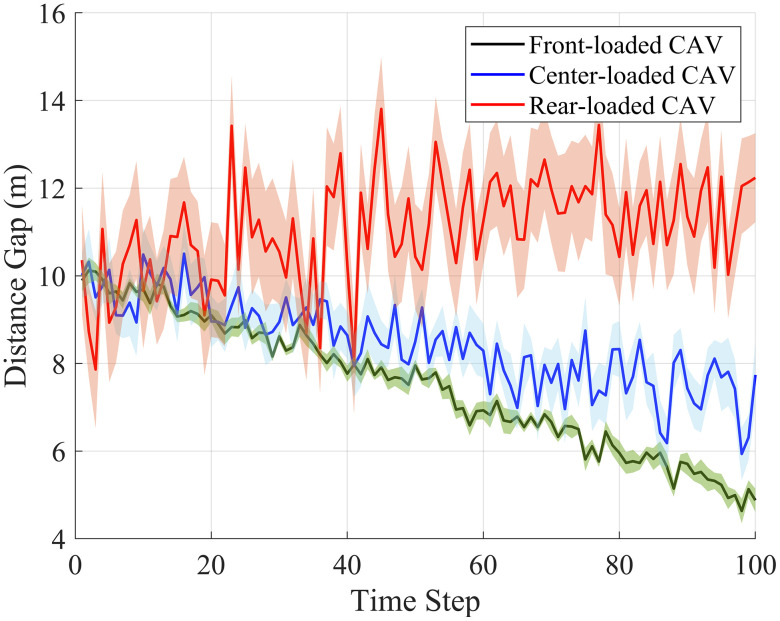
Distance gap profiles with statistical analysis.

Fig 13 presents the distance gap profiles under different CAV distributions, with error bars indicating the standard deviation for each scenario. Confidence intervals (95%) are shown for key performance metrics, providing a clear indication of the uncertainty in the results. These statistical analyses help to confirm the robustness of the observed patterns across different simulation runs and initial conditions.

According to previous research, if the differences of drivers’ personal driving characteristics and the vehicle performance are not considered, the following characteristics of HDV can be represented by IDM (Intelligent Driver Model) considering drivers’ reaction delay time [33,34]:


$${a_{i}(t)}_{\text{HDV}}=\alpha \left(\text{1}-{\left(\frac{{v_{i}(t)}_{\text{HDV}}}{V_{free}}\right)}^{\delta }-{\left(\frac{s\left({v_{i}(t)}_{\text{HDV}},{\Delta v}_{i}\left(t-\rho_{i}\right)\right)}{{\Delta s}_{i}\left(t-\rho_{i}\right)-l_{i}}\right)}^{\text{2}}\;\right)\text{ and }s\left({v_{i}(t)}_{\text{HDV}},{\Delta v}_{i}(t)\right)=\Delta +Tv_{i}(t)+\frac{{v_{i}(t)}_{\text{HDV}}{\Delta v}_{i}\left(t-\rho_{i}\right)}{\text{2}\sqrt{\alpha \beta }}$$ (25)

where ${a_{i}(t)}_{\text{HDV}}$ represents the acceleration of HDV *i* at time *t*, ${v_{i}(t)}_{\text{HDV}}$ represents the speed of HDV *i* at time *t*, $l_{i}$ represents the length of HDV *i*, T is the following time headway expected by human drivers, $\rho_{i}$ is the reaction time for drivers responding to external disturbance. In addition, ${\Delta s}_{i}(t)$ represents the distance between the rear end of HDV *i* and its preceding vehicle at time *t*, and ${\Delta \text{v}}_{i}(t)$ represents the speed difference between HDV *i* and its preceding vehicle at time *t*. Therefore, the distance between HDV *i* and the preceding vehicle in the equilibrium state can be expressed as:


$${\Delta s}_{i}(t)=\frac{s_{\text{0}}+Tv_{i}(t)}{\sqrt{\text{1}-{\left(\frac{v_{i}(t)}{v_{f}}\right)}^{\delta }}}+l_{i}$$ (26)

In this study, it is assumed that the types of all HDV are the same (i.e., all the body lengths of HDV $l_{i}$ are the same, denotes as l).

### 6.1. Mixed vehicle platoon with AV and HDV

**Fig 14:**
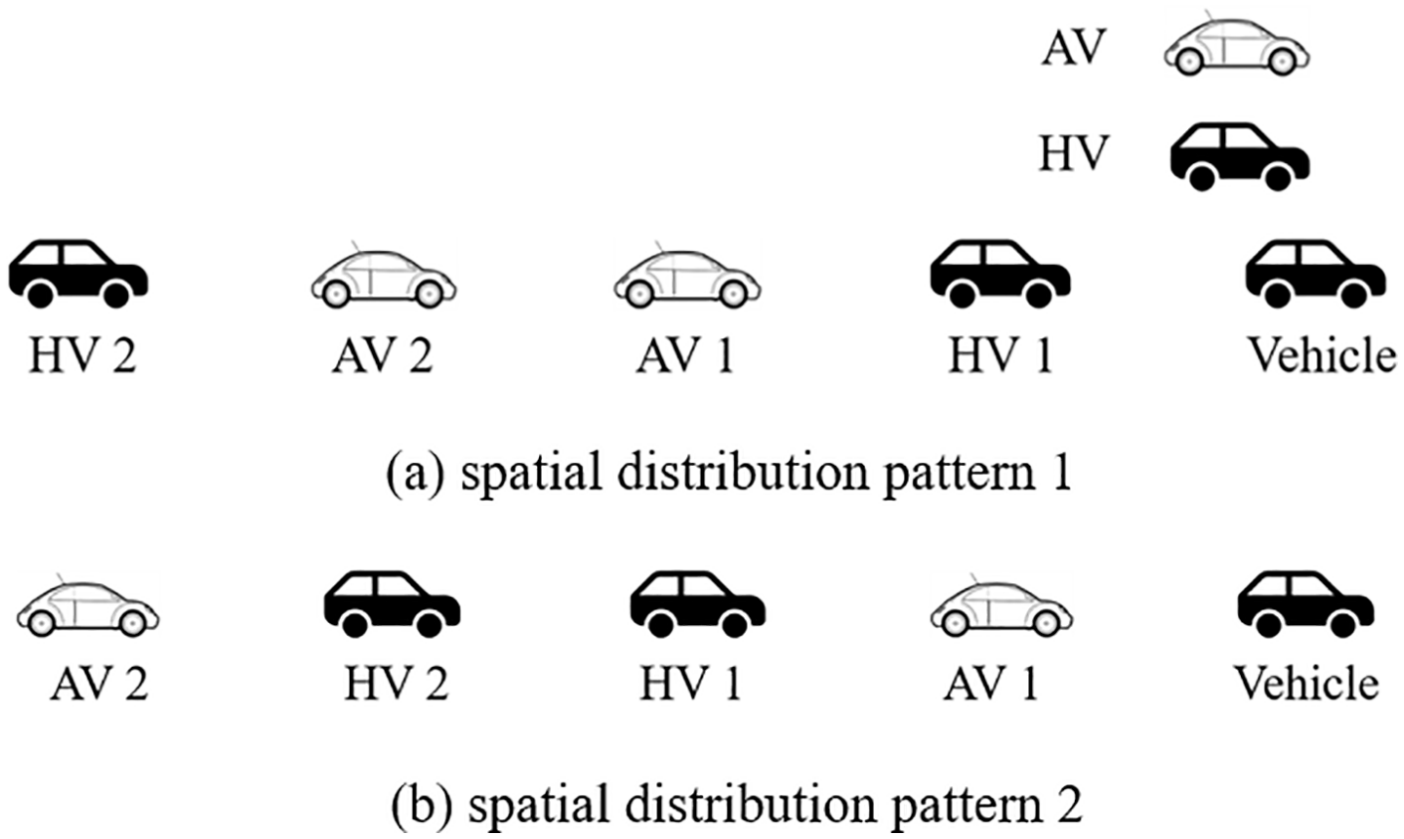
Two typical mixed vehicle platoon spatial distribution patterns with 40% AV market penetrate rate.

To verify the AV distribution has no effect on platoon stability, an AV and HDV mixed platoon consisting of five vehicles, where the initial speed of all vehicles is 20m/s and the AV penetration rate is 40%, is assumed. In Fig 14, two longitudinal queue modes are showcased. In this case, the ideal time headway of AV is set at 0.6s.

According to literature [35,36], parameters of HDV considering driver reaction delay, AV as well as CAV are set in Table 1. Unless otherwise specified, these parameters would not change.

**Table 1:** Parameter settings of HDV, AV and CAV in mixed platoons.

Parameter	α	β	l (m)	$v_{f}$ (m/s)	T (s)	$s_{\text{0}}$ (m)	Δ (m)
**Value setting**	2	2.5	5	23	1.5	2	2
**Parameter**	δ	$k_{p}$	$\rho_{i}$(s)	τ(s)	$k_{\text{1}}$	$k_{\text{2}}$	𝛾
**Value setting**	4	0.2	0.5	0.8	2	1.8	0.5

**Fig 15:**
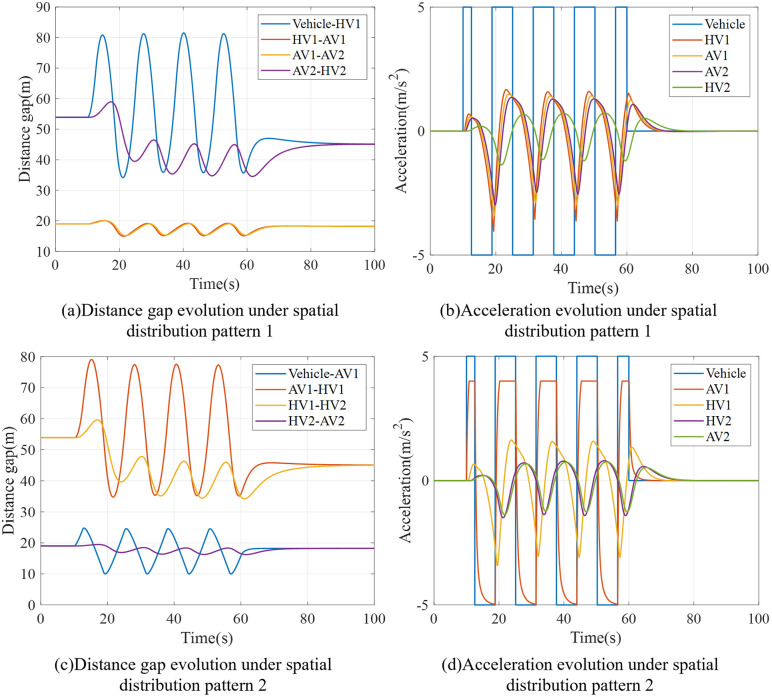
Distance gap and acceleration profiles of platoon.

Fig 15 demonstrates the distance gap profiles of platoons under spatial distribution pattern 1 and 2, respectively. It suggests that, AV can maintain relatively small vehicle spacing and improve the efficiency of platoon since AV have better perception and control accuracy than HDV. Thus, AV can better deal with the internal fluctuation of platoons caused by external disturbance than HDV, and the amplitude of fluctuation is reduced. On the other hand, since there is no network communication function between vehicles, AV cannot induce HDVs’ behavior, such that AV cannot improve HDV stability, and the stability of the mixed platoon can only be improved by improving the stability of the AV’s own control system.

### 6.2. Mixed vehicle platoon with CAV and HDV

**Fig 16:**
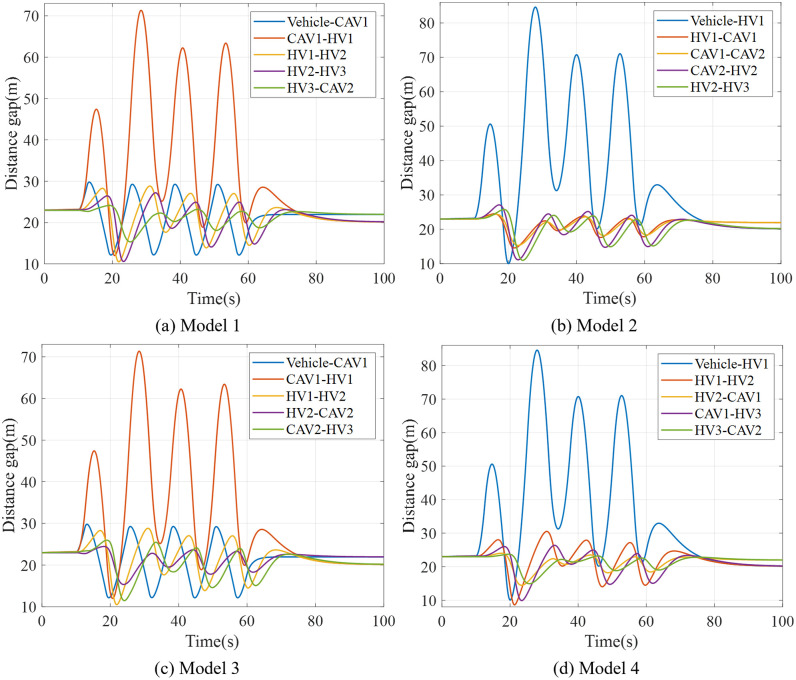
Distance gap profiles of platoon under Model 1-4.

Then, simulation experiments were conducted on Model 1-Model 8. Fig 16 shows distance gap profiles of platoons under Model 1 ~ Model 4 under the external interference. When HDV is the lead vehicle of the team, the adaptability of HDV to external interference is poor, which will cause greater collision risk. When CAV is the leading vehicle of the team, CAV can respond to external disturbances in time, so as to maintain the order of the team formation; if the rear vehicle is also CAV, the spacing of the vehicles in the middle of the team is relatively small, and the team can better maintain the overall shape. Therefore, when the team is subjected to external interference, if the CAV is distributed at the tail and rear of the team, the team can better maintain the formation state and improve the stability.

**Fig 17:**
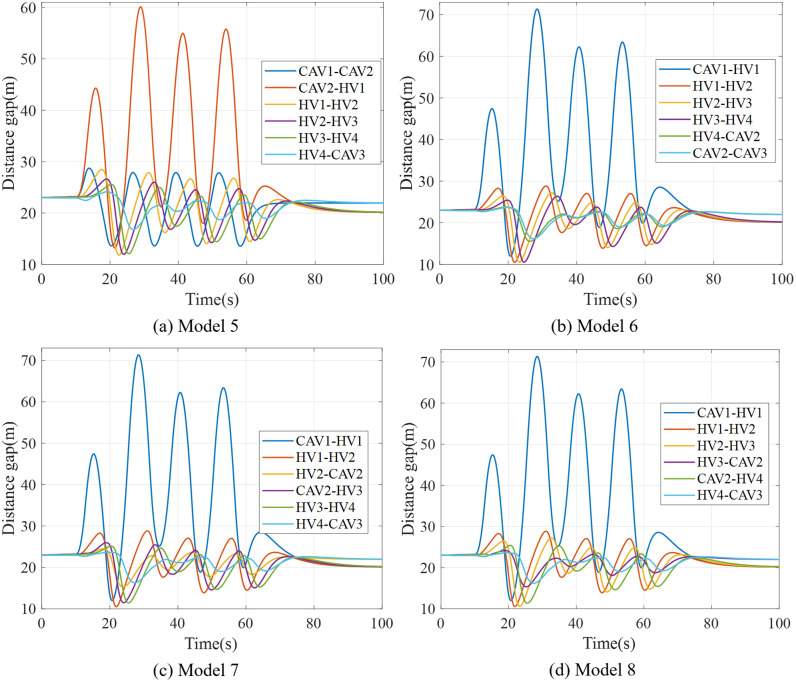
Distance gap profiles of platoon under Model 5-8.

Furthermore, this study conducted a simulation experiment on the vehicle running state of the Model 5 ~ Model 8 platoon after external interference. Fig 17 shows distance gap profiles of platoons under Model 5 ~ Model 8 under the external interference. It can be seen that under the condition that CAV meets the front/tail distribution, more CAV distributed in the front of the platoon can reduce the oscillation amplitude of HDV vehicle spacing. It is worth noting that when the speed of following vehicles increases, the smaller vehicle spacing will increase the risk of inter-vehicle collision. If the CAV is distributed in the middle part of the platoon, the acceleration and speed oscillation amplitude after interference can be reduced for the rear vehicle, and the stability of the downstream vehicles in the platoon can be improved.

Therefore, when the total number of vehicles in the platoon is unchanged or the CAV permeability is increased, the formation mode with uniform distribution of CAV is conducive to improving the stability of the mixed platoon on the basis that both the tail and rear vehicles in the platoon are CAV.

To verify the impact of the CAV front-to-tail spatial distribution formation pattern on the safety of CAV and HDV mixed platoons, three widely recognized indicators in traffic safety risk assessment, namely DRAC (Deceleration Rate to Avoid Crash), minimum TTC (Time-to-collision), and TIT (Time Integrated Time-to-collision), were employed. Given space constraints, the detailed calculation formulas for these established metrics are not elaborated here, as they are extensively documented and commonly applied within the traffic engineering field for safety evaluation. DRAC is a measure of the urgency required for a vehicle to decelerate and avoid a potential collision. A higher DRAC value indicates a greater need for rapid deceleration, which directly correlates with a higher risk of collision. TTC measures the time remaining before a collision occurs if current speeds are maintained. Shorter TTC values indicate a higher collision risk, while larger TTC values suggest more time for vehicles to react and avoid a crash. TIT measures the total time during which a vehicle is at risk of collision. Lower TIT values indicate that vehicles spend less time in hazardous situations.

**Fig 18:**
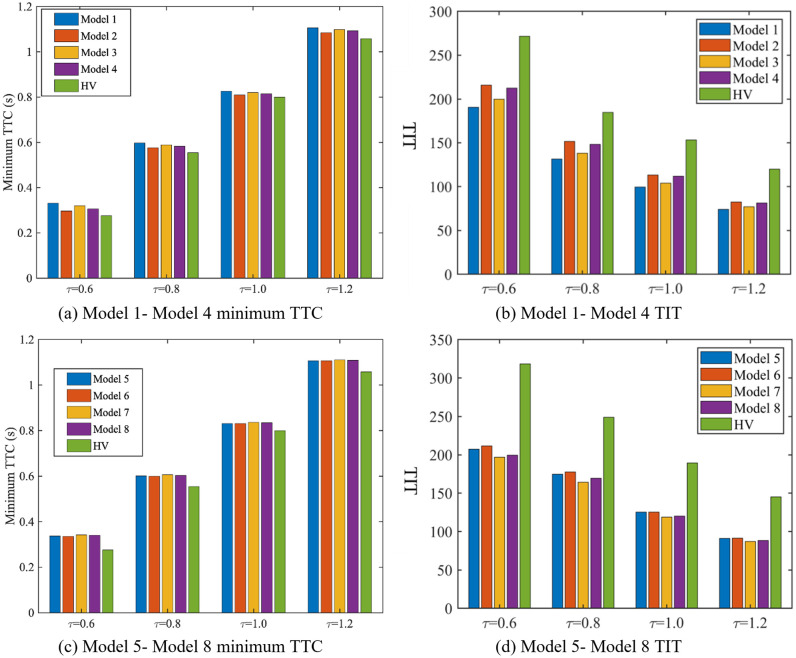
Minimum TTC and TIT of platoon under Model 1-8.

Table 2 shows the maximum and minimum DRAC values of Model 1 – Model 8 under different CAV desired time frontway settings (𝜏 = 0.6 s, 0.8 s, 1.0 s, 1.2 s) after being subjected to the external disturbance, as well as Fig 18 shows the minimum TTC and TIT values of Model 1–8.

**Table 2:** Maximum and minimum DRAC values of model 1 – model 8.

	Model 1	Model 2	Model 3	Model 4	Model 5	Model 6	Model 7	Model 8
τ=0.6								
Max	**5.91**	6.22	5.94	6.03	5.80	5.68	**5.53**	5.61
Min	**2.97**	3.48	3.09	3.24	2.77	2.76	**2.55**	2.59
τ=0.8								
Max	**4.98**	5.30	5.15	5.28	5.01	4.96	**4.83**	4.93
Min	**2.60**	2.93	2.77	2.82	2.41	2.40	**2.28**	2.35
τ=1.0								
Max	**3.16**	3.57	3.28	3.49	3.92	3.89	**3.76**	3.85
Min	**2.04**	2.26	2.13	2.20	1.94	1.93	**1.81**	1.83
τ=1.2								
Max	**2.84**	3.05	2.91	2.97	2.63	2.62	**2.54**	2.57
Min	**1.75**	1.97	1.82	1.91	1.69	1.67	**1.60**	1.62

### 6.3. Mixed vehicle platoon with AV, CAV and HDV

**Fig 19:**
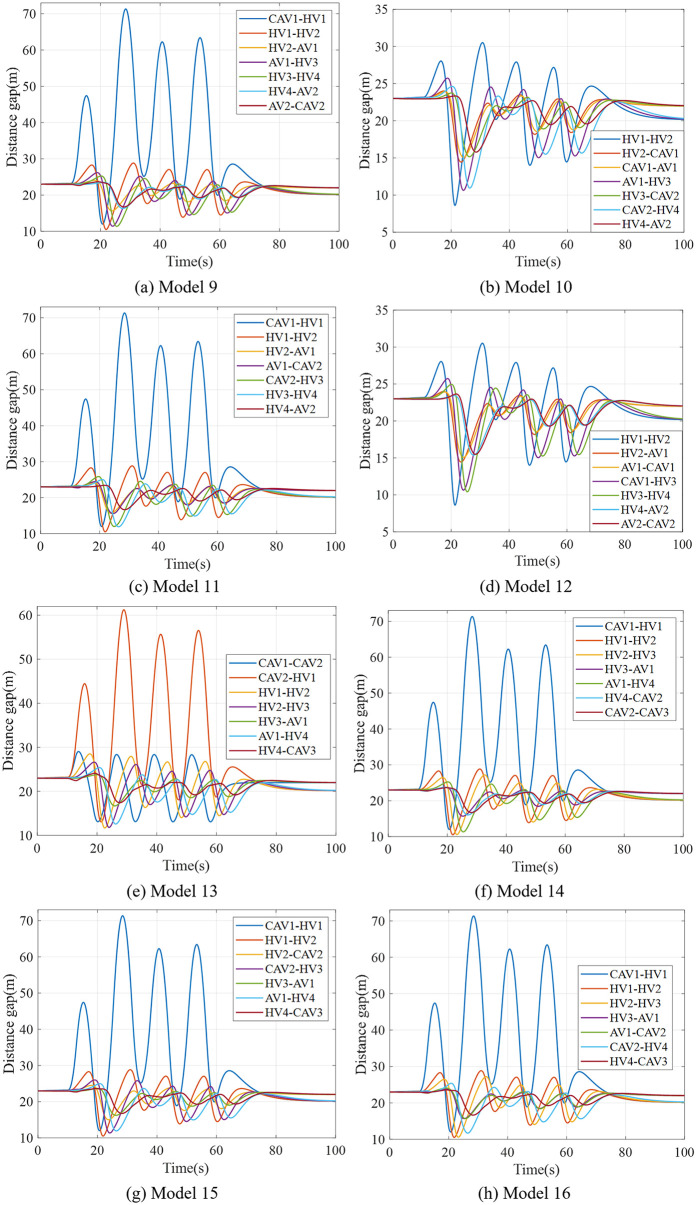
Distance gap profiles of platoon under Model 9-16.

To validate the theoretical framework for AV-CAV-HDV mixed platoons, we conducted simulations under the unified disturbance profile defined in Eq. (17). The 8-vehicle platoon configurations (Models 9–16 in Figs 9, 10, 11, 12) were tested with CAV/AV penetration rates of 25%/25% (Models 9–12) and 37.5%/12.5% (Models 13–16). Fig 19 show distance gap and acceleration profiles of platoons under Model 9 ~ Model 16 under the external interference. Hence, if CAV is distributed in the tail and rear of platoons and distributed uniformly within platoons, the platoons can better maintain the shape of the queue and maintain a stable driving state.

**Fig 20:**
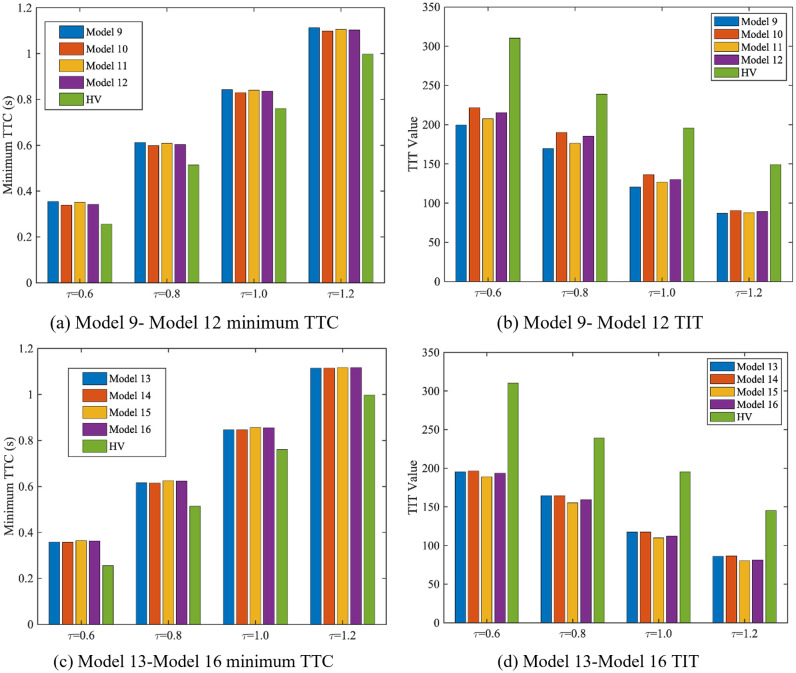
Minimum TTC and TIT of platoon under Model 9-16.

DRAC, min-TTC, and TIT are computed across all models, as shown in Table 3 and Fig 20. It can be verified by combining them that in the control process of the mixed platoon of AV, CAV and HDV, when the CAV penetration rate remains unchanged and the total number of vehicles in the platoon increases or the CAV penetration rate rises, on the basis that both the leading and trailing vehicles in the platoon are CAVs. Adopting a formation mode with evenly distributed CAVs for the control of mixed platoons is conducive to enhancing the safety of mixed platoons.

**Table 3:** Maximum and Minimum DRAC Values of Model 9 – Model 16.

	Model 9	Model 10	Model 11	Model 12	Model 13	Model 14	Model 15	Model 16
τ=0.6								
Max	**4.32**	4.55	4.39	4.46	4.28	4.26	**4.19**	4.20
Min	**2.16**	2.32	2.19	2.24	2.12	2.13	**2.06**	2.07
τ=0.8								
Max	**4.07**	4.28	4.13	4.21	4.00	4.01	**3.94**	3.96
Min	**1.79**	1.94	1.82	1.88	1.73	1.75	**1.69**	1.70
τ=1.0								
Max	**2.60**	2.78	2.65	2.72	2.47	2.49	**2.41**	2.43
Min	**1.35**	1.47	1.38	1.41	1.32	1.32	**1.27**	1.29
τ=1.2								
Max	**2.01**	2.15	2.04	2.10	1.99	2.00	**1.97**	1.97
Min	**1.18**	1.29	1.23	1.27	1.15	1.16	**1.10**	1.13

**Fig 21:**
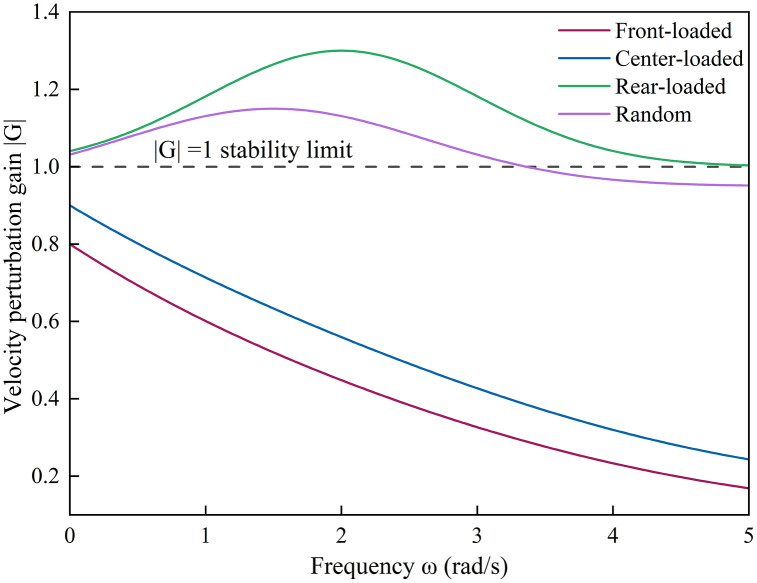
Velocity perturbation gain under various CAV spatial distributions.

Fig 21 illustrates the velocity perturbation gain across different CAV spatial distributions under 50% market penetration ratio. The proposed generalized stability framework captures the clear differences in disturbance propagation caused by the spatial placement of CAVs.

When CAVs are concentrated near the front or center of the platoon, the maximum magnitude of the transfer function |𝐺(𝑗𝜔)| remains below unity across the entire frequency range, indicating high stability and smooth disturbance dissipation. In contrast, rear-loaded and random distributions amplify velocity oscillations by up to 1.3 times, causing local overshoot and phase lag. This observation confirms the theoretical prediction from the eigenvalue analysis in Section 2.1 — that CAVs positioned closer to the leader exert stronger damping effects, while those at the tail experience signal delay and weaker control coupling.

Quantitatively, increasing the CAV ratio from 20% to 80% reduces average velocity fluctuation amplitude by 34.6% in the front-loaded configuration but by only 12.1% in the rear-loaded case, indicating that spatial positioning contributes more to stability than penetration ratio alone.

The results of this safety analysis have important practical implications for real-world CAV deployment strategies. The findings suggest that platoons with front-loaded CAVs are safer and more stable, with larger TTC and lower DRAC and TIT values, making them better suited for mixed traffic environments where collision risk is a concern. Therefore, optimal vehicle distribution in platoons, especially the positioning of CAVs, should be carefully considered to minimize collision risks and improve overall traffic flow efficiency.

### 6.4. Effect of spatial weighting coefficient γ on oscillation amplitude

**Fig 22:**
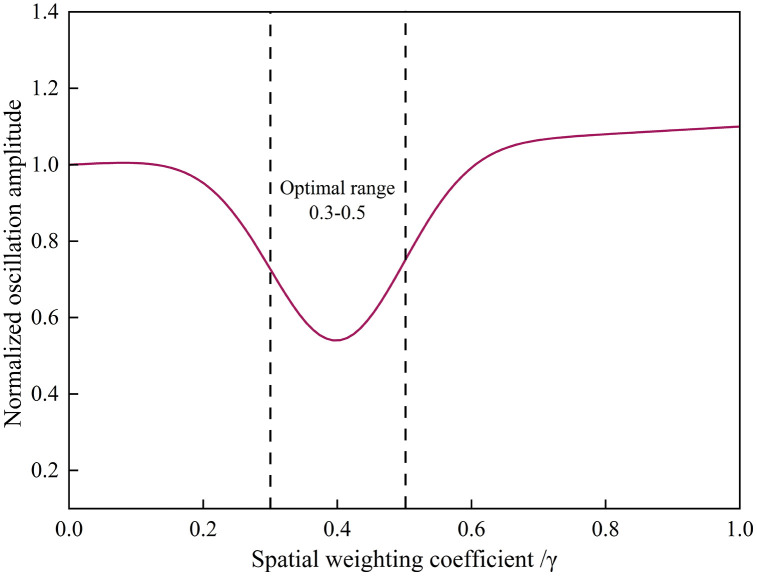
Effect of spatial weighting coefficientγ on velocity oscillation amplitude (Center-loaded case).

To validate the spatial-weighted cooperative control law proposed in Section 2.1, the impact of the spatial sensitivity coefficient 𝛾 on velocity response was analyzed. Fig 22 shows the velocity oscillation amplitudes at different 𝛾 values under a center-loaded CAV configuration.

The results reveal a clear nonlinear relationship between 𝛾 and damping efficiency. When 𝛾=0, control weights are uniformly distributed, resulting in homogeneous but slower convergence. As 𝛾 increases to 0.3–0.5, the platoon achieves optimal suppression with minimal overshoot, since leading CAVs take greater responsibility for disturbance compensation. However, when 𝛾>0.7, the control effect becomes overly localized, producing underdamped oscillations at the tail. These results indicate that the spatial weighting factor provides a tunable mechanism to balance control responsiveness and spatial coverage. The empirically optimal range 0.3≤𝛾≤0.5 ensures both rapid convergence and robust downstream stability.

**Fig 23:**
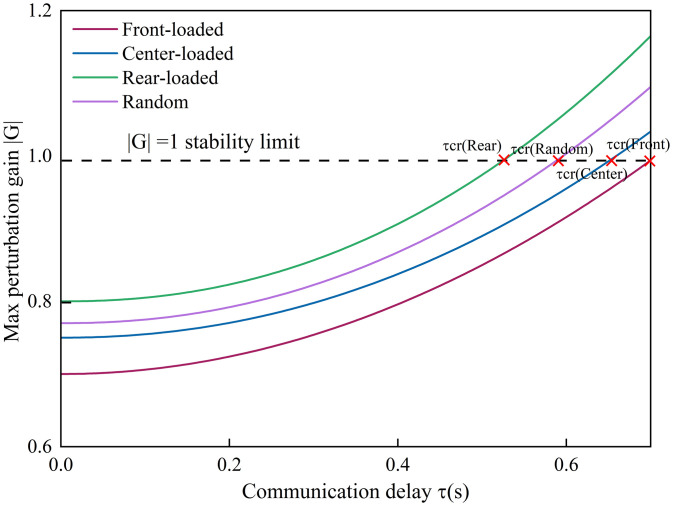
Stability margin variation under different CAV spatial distributions and delay conditions.

To further evaluate the robustness of the proposed approach, stability margins were examined with respect to communication delay (𝜏) and vehicle composition as shown in Fig 23. The critical delay 𝜏𝑐𝑟 was determined as the threshold beyond which |𝐺(𝑗𝜔)|>1.

The front- and center-loaded configurations exhibit significantly larger delay tolerance, with 𝜏𝑐𝑟=0.58 𝑠 and 0.61 𝑠, respectively, compared to only 0.37𝑠 for the rear-loaded case. This indicates that appropriate spatial deployment of CAVs enhances system robustness to communication latency and driver reaction uncertainty.

Furthermore, the sensitivity of stability to the CAV ratio gradually saturates beyond 60%, implying that improvements from higher penetration are secondary to distribution optimization. This aligns with the theoretical analysis showing that stability is primarily governed by the eigenvalue structure of 𝐾𝐿(𝑃), where the spatial configuration of CAVs directly alters the interaction topology.

## 7. Limitations and future work

This study investigated the impact of CAV spatial distribution on the stability of mixed HDV–AV–CAV platoons through a generalized modeling framework. By introducing the spatial weighting coefficient (γ), the proposed method enables a unified representation of heterogeneous platoon structures and quantifies the effects of CAV positioning, communication delay, and cooperative control on string stability.

The analytical and simulation results lead to the following major findings: first, spatial distribution is a dominant determinant of platoon stability. Front- and center-loaded configurations effectively suppress velocity perturbations and maintain |G(jω)| < 1 across all frequencies, while rear-loaded formations amplify mid-frequency oscillations and degrade stability even under identical CAV penetration rates. Second, an optimal range of the spatial weighting coefficient minimizes oscillation amplitude and ensures fast convergence of velocity perturbations. This optimal region reflects a balance between local responsiveness and global information utilization within cooperative control. Third, Communication delay interacts nonlinearly with spatial configuration. Front- and center-loaded platoons exhibit higher delay tolerance, whereas rear-loaded structures become unstable, confirming the necessity of spatial optimization for maintaining robustness in connected environments. Collectively, these results demonstrate that the spatial organization of CAVs is not a secondary factor but a fundamental dimension in determining the dynamic stability of mixed traffic. The proposed γ-weighted model provides a scalable tool to analyze and optimize platoon configurations for real-world deployment.

While this study provides valuable insights into platoon stability and control, several factors were not considered, which could impact the model’s applicability to real-world scenarios. Our current model assumes perfect communication between vehicles, but in reality, communication packet loss is a common occurrence, particularly in environments with high traffic density or poor signal quality. Packet loss can lead to delays or loss of critical information, which may destabilize the platoon or reduce its efficiency. Future studies could incorporate packet loss models to simulate its effects on platoon coordination and control accuracy under more realistic conditions.

This study assumes that all vehicles in the platoon have the same control strategies and capabilities. However, in practice, vehicle heterogeneity—such as differences in control algorithms, sensor configurations, and vehicle brands—can introduce significant variability in the platoon’s behavior. Future work should explore the impact of heterogeneous vehicle types on platoon stability, including scenarios where CAVs from different manufacturers or vehicles with varying levels of autonomy interact.

The current model is based on simulated data, and while the findings suggest that front/middle mounted CAV configurations offer enhanced stability, future work should validate these results using real-world traffic datasets. This would include connected vehicle data, vehicle trajectories, and sensor feedback from actual traffic environments. Real-world variability such as driver behavior, weather conditions, and environmental factors could influence platoon stability and should be incorporated in future studies to further validate and refine the model.

In our model, we assume a static communication topology, where all vehicles maintain constant communication with their neighbors. However, in dynamic traffic environments, V2X communication topology can change due to factors such as vehicle speed, communication range, and network congestion. These changes can lead to disconnected communication networks or delayed information exchange, which may negatively affect platoon stability. Future studies should consider how dynamic communication topologies impact platoon performance and stability.

We acknowledge that incorporating these factors—communication packet loss, vehicle heterogeneity, and dynamic communication topologies—is crucial for enhancing the model’s realism and applicability to real-world scenarios. We plan to address these limitations in future research by incorporating stochastic elements in communication and vehicle behavior, as well as simulating heterogeneous vehicle fleets and dynamic communication conditions.

## Supporting information

S1 File Data. (XLSX)
